# Universal nomenclature for oxytocin–vasotocin ligand and receptor families

**DOI:** 10.1038/s41586-020-03040-7

**Published:** 2021-04-28

**Authors:** Constantina Theofanopoulou, Gregory Gedman, James A. Cahill, Cedric Boeckx, Erich D. Jarvis

**Affiliations:** 1 Laboratory of Neurogenetics of Language, Rockefeller University, New York, NY USA; 2 Section of General Linguistics, University of Barcelona, Barcelona, Spain; 3 University of Barcelona Institute for Complex Systems, Barcelona, Spain; 4 ICREA, Barcelona, Spain; 5 Howard Hughes Medical Institute, Chevy Chase, MD USA

**Keywords:** Evolutionary genetics, Phylogenetics, Comparative genomics

## Abstract

Oxytocin (OXT; hereafter OT) and arginine vasopressin or vasotocin (AVP or VT; hereafter VT) are neurotransmitter ligands that function through specific receptors to control diverse functions^1,2^. Here we performed genomic analyses on 35 species that span all major vertebrate lineages, including newly generated high-contiguity assemblies from the Vertebrate Genomes Project^3,4^. Our findings support the claim^5^ that *OT* (also known as *OXT*) and *VT* (also known as *AVP*) are adjacent paralogous genes that have resulted from a local duplication, which we infer was through DNA transposable elements near the origin of vertebrates and in which *VT* retained more of the parental sequence. We identified six major oxytocin–vasotocin receptors among vertebrates. We propose that all six of these receptors arose from a single receptor that was shared with the common ancestor of invertebrates, through a combination of whole-genome and large segmental duplications. We propose a universal nomenclature based on evolutionary relationships for the genes that encode these receptors, in which the genes are given the same orthologous names across vertebrates and paralogous names relative to each other. This nomenclature avoids confusion due to differential naming in the pre-genomic era and incomplete genome assemblies, furthers our understanding of the evolution of these genes, aids in the translation of findings across species and serves as a model for other gene families.

## Abstract (web summary)

A revised, universal nomenclature for the vertebrate genes that encode the oxytocin and vasopressin–vasotocin ligands and receptors will improve our understanding of gene evolution and facilitate the translation of findings across species.

## Main

OT and VT act as hormones or neurotransmitters that—through their respective G-protein-coupled receptors—regulate a wide range of biological functions, including uterine contractions and milk ejection in placental mammals; copulation, bond formation, thermoregulation, nesting behaviour and social vocalizations (for oxytocin) across many vertebrate and some invertebrate groups; and antidiuresis, blood pressure, parental care and reproduction (for vasotocin) in mammals and/or other vertebrates and invertebrates^1,2^ (Supplementary Table 1, Supplementary Note 1). In the pre-genomic era, small differences in amino acids of the OT and VT hormones in different species or lineages led biochemists to give them and their receptors different names: for example, mesotocin in birds, reptiles and frogs, and isotocin in teleost fish, for the apparent oxytocin complement of mammals; and vasopressin in mammals for the apparent vasotocin complement in other vertebrates^6^.

It has previously been hypothesized that *OT* and *VT* are the product of a local duplication near the origin of vertebrates^5^. However, the evolutionary trajectory of the receptors is under debate^7–10^. One recent view^9^ is that the genes that encode the OT and VT receptors (hereafter, OTR-VTRs) evolved through two rounds of whole-genome duplication in the ancestor of cyclostomes. An alternative view^10^ posits that the OTR-VTRs evolved by one round of whole-genome duplication shared by agnathans and gnathostomes, followed by segmental duplications. However, these studies used highly fragmented genome assemblies and inconsistent annotations, and could not conclusively resolve the evolution of the OTR-VTRs. The resulting varied biochemical-based and evolutionary-based terminologies have led to confusion as regards the orthology and paralogy of these genes, which is emblematic of a wider problem in gene nomenclature.

Here we analysed the genomes of 35 species that span all the major vertebrate lineages as well as an additional 4 outgroup genomes from invertebrate lineages (Supplementary Table 2); these included several species that were sequenced with long-read and long-range scaffolding technologies by the Vertebrate Genomes Project (VGP) (https://vertebrategenomesproject.org/), which filled gaps and corrected errors of previous shorter-read assemblies^3^. On the basis of gene synteny, sequence identity, family tree and other analyses, we propose that *OT* and *VT* are paralogous genes that arose through a local duplication via DNA transposable elements near the origin of vertebrates. We propose that the OTR-VTR genes evolved by a combination of whole-genome duplication and segmental duplication, which led to six receptors near the origin of jawed vertebrates with lineage-specific losses and gains thereafter. With this improved understanding of the relations between the OTR-VTR genes, we propose a universal vertebrate nomenclature based on evolutionary relationships (Table 1).

**Table 1:** Previous and proposed terminology for genes encoding OT and VT ligands and receptors in vertebrates

**Mammals**	**Birds**	**Turtles and crocodiles**	**Frogs**	**Fish**	**Sharks**	**Universal vertebrate revision**
Oxytocin (*OXT*, *OT*, *Oxy*) Neurophysin (*NPI*) Mesotocin (*MT*)	Mesotocin (*MT*, *MST*) Oxt-like Neurophysin-1-like	Mesotocin (*MT*, *MST*)	Mesotocin (*MT*, *MST*)	Mesotocin (*MT*) Isotocin (*IT*, *IST*) Glumitocin Neurophysin *IT-1*-like, *IT-NP*	Valitocin Aspargtocin	Oxytocin (*OT*)
Arginine vasopressin (*AVP*, *ARVP*, *AVRP*, *Vp*, *Vsp*) Neurophysin II (*NP2*) Lysine vasopressin Phenypresin	Vasotocin (*VT*)	Vasotocin (*VT*)	Vasotocin (*VT*)	Vasotocin (*VT*) *VT-NP*, *avpl*, *vsnp*	Vasotocin (*VT*)	Vasotocin (*VT*)
*OXTR*, *OTR*	*VT3*, *MTR*	*OXTR*	*MesoR*, *OXTR*	*ITR*, *OXTR*, *itnpr*-like 2, *itr2*	*OXTR*	Oxytocin receptor (*OTR*)
*AVPR1a*, *V1aR*, *V1A*	*VT4*, *VT4R*		*Avpr1*, *VasR*	*Avpr1aa*, *VasR*, *Avpr1ab*		Vasotocin receptor 1A (*VTR1A*, *V1A*)
*AVPR1b*, *V1bR*, (*A*)*VPR3*, *V3*, *VIBR*	*VT2*, *AVT2R*					Vasotocin receptor 1B (*VTR1B*, *V1B*)
	*VT1*, *AVPR2*		*Avpr2*.2	*V2C*, *V2bR2*, *Avpr2*.2, *V2L*	*V2C*, *V2bR2*	Vasotocin receptor 2A (*VTR2A*, *V2A*)
				*V2B*, *V2BR1*, *V2RI*, *OTRI*, *nft*, *avpr2*		Vasotocin receptor 2B (*VTR2B*, *V2B*)
*AVPR2*, *V2R*, *VPV2R*				*Avpr2bb*, *V2A*(*2*), *avpr2a*(*a*)		Vasotocin receptor 2C (*VTR2C*, *V2C*)

Long (for example, *VTR1A*) and short (for example, *V1A*) versions of the gene symbols are given. Aliases include terminology in the NCBI gene database. A complete list of aliases can be found in Supplementary Table 4a–e.

## Approach

In all genomes, we initially searched for *OT*, *VT* and OTR-VTR genes using pair-wise BLAST and BLAT analyses, and analysed the synteny of these genes from microchromosomal to macrochromosal scales between and within species. We then assessed congruence between synteny, sequence identity and gene family trees.

## Evolution of the VT and OT ligands

**Fig. 1:**
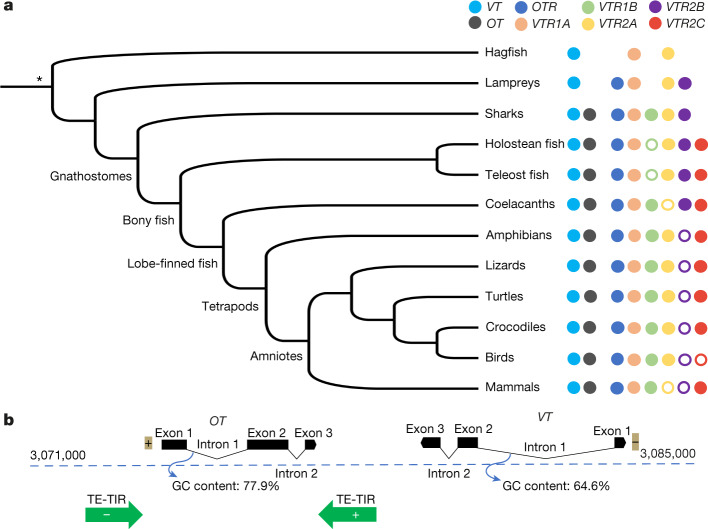
Phylogenetic distribution and local gene duplication. **a**, Phylogenetic distribution of *OT*, *VT* and OTR*-*VTR genes among vertebrates. Filled circles, presence of a gene; empty circles, loss of a gene; no circle, the gene never evolved in that lineage. Phylogenetic tree based on ref. ^36^. *Unresolved relationship for whether hagfishes and lampreys constitute a single phylum or two separate phyla^35,37^. **b**, Local chromosomal organization of the *OT* and *VT* region. Representation of the position (in kb), orientation (+ or −) of *OT* and *VT* genes (exons + introns) in human chromosome 12, intron length (scale, 100 bases), GC content and DNA transposable elements with terminal inverted repeats (TE-TIRs) (green).

**Extended Data Fig. 1:**
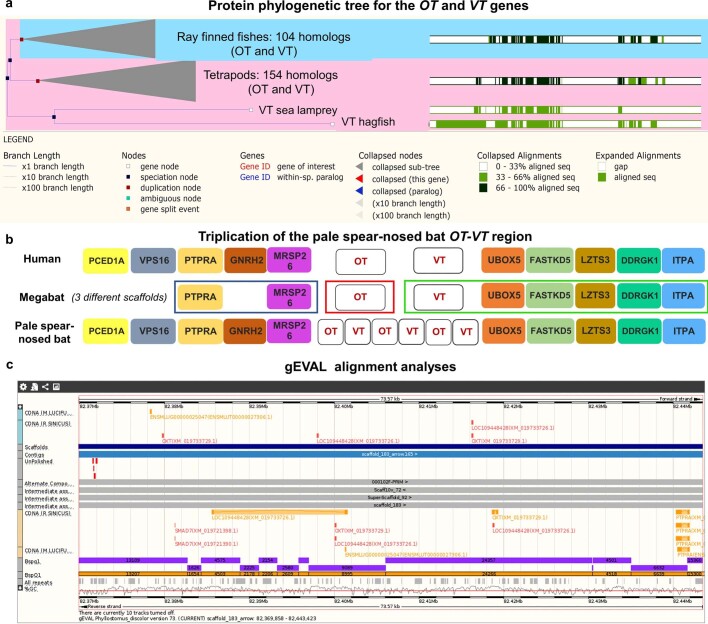
Lineage specific *OT-VT* specializations. **a**, Protein phylogenetic tree for *VT* in hagfish and lamprey relative to other vertebrates. Maximum likelihood amino acid phylogenetic tree generated via the Ensembl ‘Gene tree’ tool (gene tree identifier: ENSGT00390000004511) that uses the Gene Orthology/Paralogy prediction method pipeline. The longest available protein of each species was used. The tree is reconciled with a species tree, generated by TreeBeST. Left, red boxes, inferred gene duplication node; blue boxes, inferred speciation events; turquoise boxes, ambiguous nodes. Right, green bars, multiple amino acid alignment made with MUSCLE; white areas, gaps in the alignment; dark green bars, consensus alignments. We curated the tree and renamed genes using the universal nomenclature proposed in this Article. The tree with the current nomenclature used in the annotations of these genomes can be found at http://www.ensembl.org/Multi/GeneTree/Image?collapse=none;db=core;gt=ENSGT00390000004511. **b**, Triplication of the pale spear-nosed bat *OT-VT* region. An approximately10-gene window of synteny between human, megabat and pale spear-nosed bat is shown. In megabat, *OT*, *VT* and their syntenic genes are found in three different scaffolds (three boxes). In the pale spear-nosed bat with a higher-quality assembly, a syntenic triplication of the *OT-VT* region is found. **c**, gEVAL alignment analyses (https://geval.sanger.ac.uk/index.html). This panel shows gapless Pacbio-based long-read contigs (dark blue) and gapless Bionano optical maps (yellow), which span through the entire region with the *OT* and *VT* duplications in the pale spear-nosed bat, without any noticeable assembly errors.

On the basis of BLAST searches, sequence identity and manual microsynteny analyses within a ten-gene window (microchromosomal), we found the human *VT* orthologue (that is, *AVP*) in all vertebrates analysed (Fig. 1a, Supplementary Fig. 1, Supplementary Table 3). Only the putative *VT* in hagfish did not have genes in synteny with any other vertebrate (presumably owing to the fragmented assembly), but the gene tree of this putative *VT* formed an immediate node with the lamprey *VT* (Extended Data Fig. 1a)—which suggests it is the *VT* homologue. In jawed vertebrates (after the divergence of the lamprey and hagfish), we found the *OT* orthologue directly adjacent to *VT* except in teleost fish (Supplementary Fig. 1, Supplementary Table 4a). In teleosts, *OT* was translocated nearby on the same chromosome (or to a separate chromosome in zebrafish; Supplementary Table 4a), which supports more rearrangements in teleosts^11^. The spotted gar—which represents the divergence of the holosteans, sister to the teleosts—had both *OT* and *VT* together in the translocated *OT* region found in teleosts, indicating that there was first a translocation and then a relocation of *VT* in teleosts near its original location. A previous short-read assembly of the megabat had *OT* as the only gene on a scaffold, indicating a fragmented assembly. The pale spear-nosed bat assembly from the VGP^3^ and Bat1K project^4^ revealed a local triplication of the *OT* and *VT* genes. We found support for this triplication using single Pacbio long reads and Bionano optical maps that spanned the entire region (Extended Data Fig. 1b, c, Supplementary Note 2a): such duplications are known to be hard to assemble with short reads^12^. An *OT* orthologue was not found in lampreys and hagfish, which provides support for a previous result in lamprey^13^. This previous report was inconclusive owing to the fact that the assembly was generated from the sized-down, programmed and rearranged somatic genome, whereas we analysed a long-read germline genome of the sea lamprey^14^; the inshore hagfish data are from a short-read germline genome.

In three of the four invertebrate species we analysed, we identified a single gene that was structurally similar (3–4 exons)—but not syntenic—to the vertebrate *VT* (Supplementary Table 5), supporting previous findings^15–17^. The exception was amphioxus, which had three copies of the *VT* gene: two on the same scaffold 23 kb apart from each other, and the other on another scaffold in a paralogous syntenic territory (Supplementary Table 5, Supplementary Note 2b). Two of these three genes had previously been noted^13,18^, which—together with our data—indicates several lineage-specific duplications in amphioxus.

To test the hypothesis that vertebrate *OT* could be a tandem duplication of *VT*^5^, we searched for DNA transposable elements, which are known to drive gene duplications^19^. We found transposable elements around *OT* (for example, in human and chimpanzee), but not around *VT* (Fig. 1b, Supplementary Tables 6, 7). These transposable elements had terminal inverted repeats, which are known to transpose through a cut-and-paste mechanism that creates an extra copy at the donor site^19^. We searched for other features that are encountered in duplicated genes, such as intron shortening and/or an increase in GC content^20^: both of the human *OT* introns were shorter than the *VT* introns, with the first *OT* intron also being 13% richer in GC content (77.9% versus 64.6%) (Fig. 1b). These relationships varied among species, with the elephant shark—representing a more basal vertebrate divergence than that of human—showing a large decrease in length of only the first *OT* intron compared to *VT* (3,226 bp versus 1,158 bp) but similar GC content.

We also found that the orientation of the genes was tail-to-head *OT*-to-*VT* (same direction) in nearly all vertebrates (including marsupial mammals)—except for placental and monotreme mammals, in which the orientation was tail-to-tail (*OT* inverted) (Supplementary Table 8). This is indicative of the fact that, after the original *OT* tandem duplication of *VT*, *OT* inversions either occurred independently at the origin of monotremes and placental mammals (as previously suggested^13^) or occurred at the origin of mammals with marsupials reverting back to the tail-to-head orientation. We also identified an independent *OT* inversion in the spotted gar (Supplementary Table 8). The totality of our findings suggest that *OT* is a local tandemly duplicated copy of *VT* that arose after the divergence of jawed vertebrates, which was followed by divergences in introns, GC content, gene orientation, translocations and further duplications in different lineages.

## A universal nomenclature for *OT* and *VT*

On the basis of these findings, we propose a universal nomenclature in which oxytocin (that is, *OT*) and vasotocin (that is, *VT*) are used for these genes in all jawed vertebrates, and *VT* is used in all jawless vertebrates and closely related invertebrates. We believe that these genes should be named in this manner because it portrays their evolutionary history, as is standard practice for other genes that are orthologous across species (for example, *FOXP1*) and paralogous within species (for example, *FOXP2*, *FOXP3* and *FOXP4*). According to this practice, the genes encoding these two peptides would be named vasopressin 1 (*AVP1*) and vasopressin 2 (*AVP2*), vasotocin 1 (*VT1*) and vasotocin 2 (*VT2*) or oxytocin 1 (*OT1*) and oxytocin 2 (*OT2*). As we realize that this would be a far-reaching shift from the existing nomenclature, we propose that the common origin of these genes be portrayed through the shared suffix -tocin, and paralogy conveyed through different root words oxy- and vaso-. Vasotocin is a name that is already used by most scientific communities focusing on non-mammalian species (Table 1). Furthermore, the name ‘arginine vasopressin’ (*AVP*) entails that this gene encodes an arginine as the eighth amino acid, which is not the case for all mammals^6^. For non-mammalian species, this means that the peptides currently known as mesotocin, isotocin, glumitocin, valitocin, aspargtocin and neurophysin in different lineages would now be called by one orthologous name (that is, oxytocin) (Table 1).

## Six vertebrate OTR–VTRs

Our manual microsynteny analysis within a ten-gene window revealed six paralogous receptors among vertebrates (Fig. 1a, Supplementary Tables 3, 4b–e). Most vertebrate species had four or five of the six receptors, and some had further lineage-specific duplications (Extended Data Fig. 9, Supplementary Note 2c–e). For greater clarity, we present our findings using our proposed nomenclature of the root names for the ligands, with evolution-based suffixes (Table 1) from evidence highlighted in the ‘Evolution of the VT and OT ligands’ section.

**Fig. 2:**
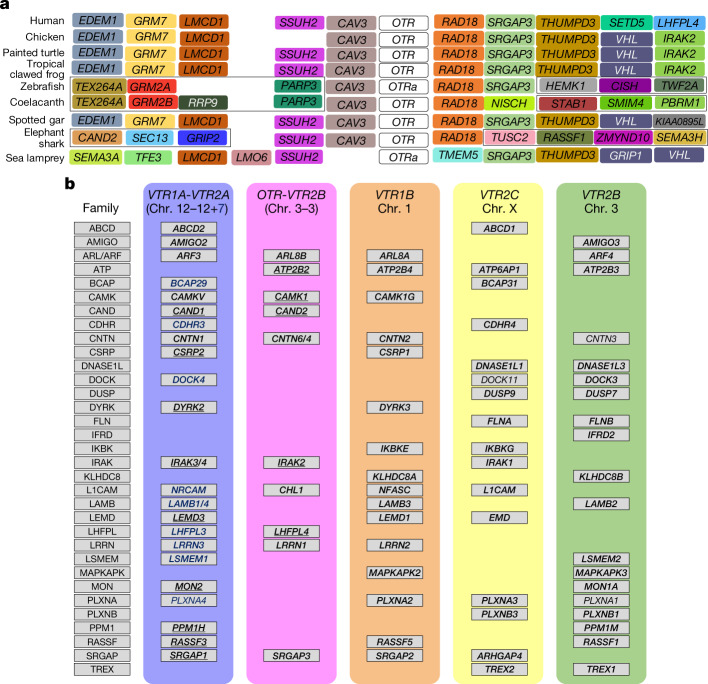
Interspecies and intraspecies synteny analyses. **a**, Example of interspecies ten-gene microsynteny for *OTR* across vertebrates. Same colour, orthologous genes. Black boxes, genome rearrangements. *OTRa* in the sea lamprey and zebrafish is orthologous to *OTR* in all other vertebrates. Human *OTR* is currently known as *OXTR*; tropical clawed frog *otr* is currently known as *oxtr*. **b**, Intraspecies 10-Mb macrosynteny among 6 chromosomes (block colours) for all OTR-VTR gene regions in humans whether present (*OTR*, *VTR1A*, *VTR1B* and *VTR2C*) or deleted (*VTR2A* and *VTR2B*). Gene families are listed alphabetically on the left. In the blue column, underlined genes were found within a 10-Mb window of *VTR1A* on chromosome 12. In the pink column, underlined genes were found within a 10-Mb window of *OTR* on chromosome 3; genes in black bold were found within a 10-Mb window of the deleted *VTR2A* on chromosome 12 or (in blue bold) 7, or within the 10-Mb window of the deleted *VTR2B* on chromosome 3. Orange column, all genes listed (in black bold) were found within a 10-Mb window of *VTR1B* on chromosome 1 (orange block). Yellow column, all genes listed (in black bold) were found within a 10-Mb window of *VTR2C* on chromosome X (yellow block). Green column, an alternative syntenic territory of *VTR2B* (green) was also found at a different location of chromosome 3. Genes not in bold are found outside of the strict 10-Mb window, but are on the same chromosome as the respective OTR-VTR gene.

On the basis of microsynteny analyses, we found the gene that is commonly defined as the oxytocin receptor (*OXTR*) in mammals or the mesotocin receptor (*MTR*) in birds (henceforth referred to as *OTR*) in a well-conserved syntenic region in nearly all of the vertebrates we examined (Fig. 2a, Supplementary Fig. 2). We found similar results for the gene known as arginine vasopressin receptor 1 (*AVPR1*) or arginine vasopressin receptor 1A (*AVPR1A*) in mammals, or vasotocin receptor 4 (*VT4*) in some non-mammals (henceforth referred to as vasotocin receptor 1A (*VTR1A*)) (Supplementary Fig. 3). By contrast, the gene known as *AVPR3* or *AVPR1B* in mammals or as vasotocin receptor 3 (*VT3*) in non-mammals (henceforth referred to as vasotocin receptor 1B (*VTR1B*)) was present in all tetrapods, sharks and coelacanths, but was absent in other fish and lampreys (Supplementary Fig. 4). The syntenic territory of *VTR1B* was present in all fish except lampreys, which indicates a gain of *VTR1B* after the divergence from lampreys that was followed by a loss in holosteans and teleosts after their divergence from coelacanths and sharks (Fig. 1a). In addition, teleosts showed rearranged syntenic gene blocks on each side of *OTR*, each side of *VTR1A* and on one side of the (lost) *VTR1B* (Supplementary Figs. 2–4).

The gene known as vasotocin receptor 1 (*VT1*) in birds, and by several other names in other lineages (Table 1) (henceforth referred to as vasotocin receptor 2A (*VTR2A*)), was found in conserved synteny in reptiles, mammals and some fish, although its syntenic territory was present in all of these lineages (Supplementary Fig. 5)—indicating independent losses (Fig. 1a). The gene known as arginine vasopressin receptor 4 (*AVPR4*) in fish (henceforth referred to as vasotocin receptor 2B (*VTR2B*)) was detected only in fishes and lampreys but its syntenic territory was detected in all vertebrates (Supplementary Fig. 6), which indicates a loss in the tetrapod ancestor (Fig. 1a). The gene commonly known as arginine vasopressin receptor 2 (*AVPR2*) in mammals or as *AVPR2A* in fishes (henceforth referred to as vasotocin receptor2 C (*VTR2C*)) was found in all vertebrates except for lampreys, elephant sharks and birds (Supplementary Fig. 7). In birds, the absent *VTR2C* was part of a larger block of about 20 genes that has been deleted^21^. These findings indicate a gain of *VTR2C* in vertebrates after the divergence of elephant sharks, followed by a loss in birds (Fig. 1a). Again, teleosts showed rearranged syntenies on either side of *VTR2B* and on one side of *VTR2A* and *VTR2C* (Supplementary Figs. 5–7).

**Extended Data Fig. 2:**
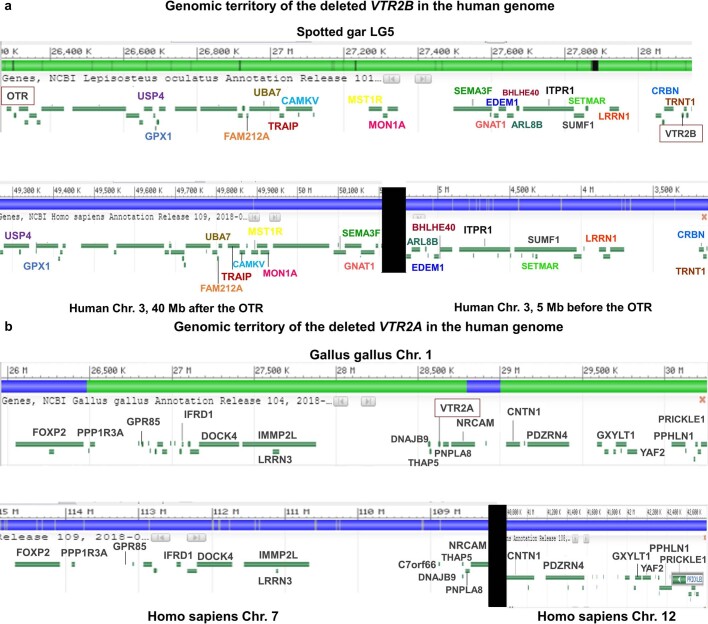
Lost VTR receptors in human, representative of mammals. **a**, Genomic territory of the deleted *VTR2B* in the human genome. The genomic territory before the spotted gar *VTR2B* (top) was found in human chromosome 3 (49–51 Mb), 40 Mb after the location of human *OTR* (bottom). The genomic territory before the spotted gar *VTR2B* was also found in human chromosome 3 (3–5 Mb), 5 Mb before the location of human *OTR*. Text colours denote orthologous genes. Solid black region links two different regions on chromosome 3 in the human genome. **b**, Genomic territory of the deleted *VTR2A* in the human genome. The genomic territory before the chicken *VTR2A* (top) was found in human chromosome 7 (100–115 Mb) (bottom). The genomic territory after the chicken *VTR2A* was found in human chromosome 12 (40–43 Mb). The solid back region links two regions from chromosomes 7 and 12 in the human genome.

**Extended Data Fig. 3:**
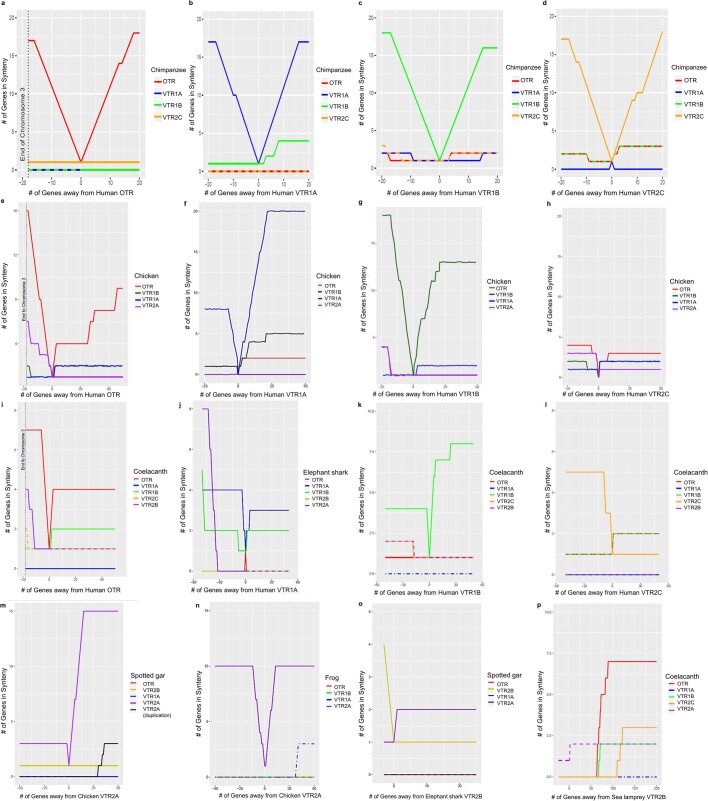
Macrosynteny SynFind analyses. **a**–**d**, Comparisons between closely related species (human and chimpanzee) for four receptors, showing maximum syntenies found using this method. **e**–**h**, Comparisons between intermediately related species (human and chicken) for the same four receptors in **a**–**d**. **i**–**l**, Comparisons between distantly related species (human and fish). **m**–**p**, Comparisons between distantly related non-human species. On the *x* axis, 0 represents the query OTR-VTR in the query organism and the numbers represent the number of genes on the 5′ (left) and 3′ (right) of the query OTR-VTR in the genome. The *y* axis shows the cumulative number of the matched homologous (orthologous or paralogous) syntenic genes in the reference genome for each reference receptor. For example, in **a** the chimpanzee *OTR* region (red line) shows 17 syntenic gene matches within 20 genes 5′ (left) of human *OTR*, and 18 matches within 20 genes 3′ (right) of human *OTR*. If the reference OTR-VTR does not show any match, then it is 0 on the *y* axis (for example, the chimpanzee *VTR1B* (shown in green in **a**)); if the reference OTR-VTR matches only the query OTR-VTR, it reaches 1 (for example, chimpanzee *VTR1A* (shown in in blue in **d**) was orthologous only to human *VTR2C*). If the reference OTR-VTR is not orthologous to the query OTR-VTR, but does show gene matches in the neighbouring territory, then it indicates a deletion of the receptor in the query species (for example, chicken *VTR1A* (shown in blue in **f**)).

In all of the species we assembled to chromosomal resolution, *OTR* and *VTR2B* were syntenic on the same chromosome and separated by 10–30 genes (Extended Data Fig. 2a, Supplementary Table 4b). Similarly, *VTR1A* and *VTR2A* were also on the same chromosome or scaffold and separated by 4–50 genes, except in mammals and fish (Supplementary Table 4c). In mammals, the syntenic genes (including *VTR1A*) on one side of the deleted *VTR2A* were on chromosome 12 (human nomenclature), whereas those on the other side were on chromosome 7^8,9^ (Extended Data Fig. 2b, Supplementary Table 4c), which indicates a fission that possibly involved the loss of *VTR2A* in mammals. In fish, there were complex patterns of rearrangements and duplications but some species (for example, the three-spined stickleback, gar, coelacanth and elephant shark) still contain *VTR1A* and *VTR2A* on the same chromosome (Supplementary Table 4c), which indicates lineage-specific chromosomal fissions and other rearrangements. In hagfish, we found only two VTR genes. These genes are located on two different scaffolds, one containing *VTR1* and the other containing *VTR2*, and each is equally syntenic for gene families containing the *OTR* and *VTR2B* combination and the *VTR1A* and *VTR2A* combination in other vertebrates (Supplementary Table 4f, g); this indicates an ancestral relationship to both chromosome combinations, possibly via duplication. Higher-quality germline assemblies for hagfish should reveal whether these two scaffolds are really separate or are part of the same chromosome. Finally, *VTR1B* and *VTR2C* were singly found on different chromosomes or scaffolds in all species in which they were present (Supplementary Table 4d, e). We verified these findings with an independent, automated, more-quantitative and longer-range measure by using SynFind^22^ on alignments in up to 100-gene macrosynteny windows around the receptors, in all major lineage combinations (Extended Data Fig. 3, Supplementary Note 3).

## Chromosomal orthology and paralogy of OTR-VTRs

**Fig. 3:**
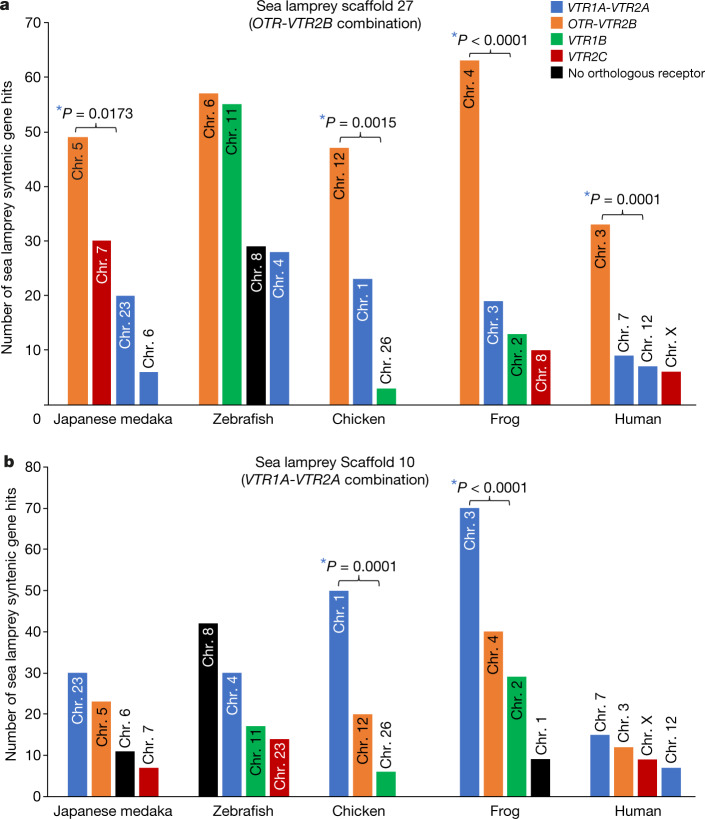
Analysis with SynMap2 identified syntenic gene hits between sea lamprey scaffolds containing two receptors each and chromosomes of other species. Bar graphs were created from dot plots. **a**, Sea lamprey scaffold 27 is most syntenic with chromosomes of other species that contain the *OTR-VTR2B* combination. **b**, Sea lamprey scaffold 10 is most syntenic with chromosomes of other species that contain the *VTR1A-VTR2A* combination. The minimum number of aligned homologous gene pairs to be considered syntenic was 3 at a 20-gene maximum distance in each species. For comparisons including human, the minimum number was set to 2. *Significant differences between chromosomes with the highest number of gene hits within a species (*P* < 0.05; *χ*^2^ test, two-sided; *n* = 199 genes located on scaffold 27; *n* = 246 genes located on scaffold 10).

**Extended Data Fig. 4:**
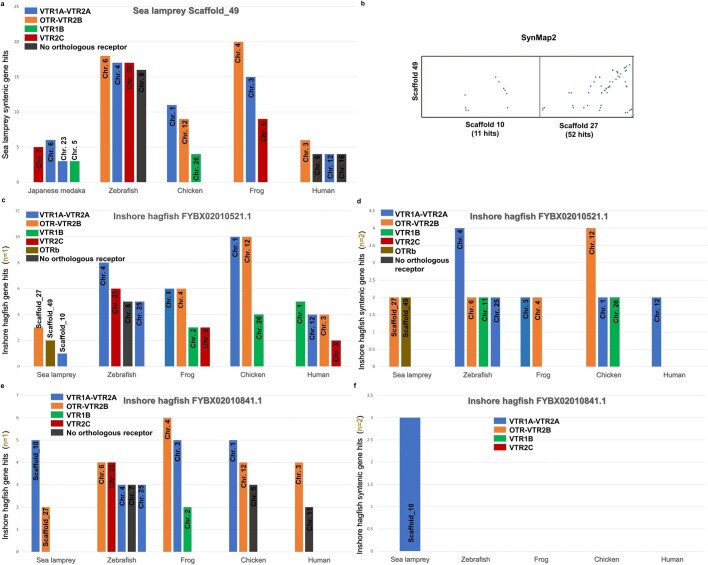
Additional chromosomal SynMap2 analyses with lamprey and hagfish. Bar graphs were created from dot plots. **a**, Additional scaffold in sea lamprey (scaffold 49) with a *VTR* gene is most syntenic with chromosomes of other species containing a *VTR1-VTR2* combination, with a 3-gene minimum per 20-gene window criterion. **b**, SynMap2 dot plot between sea lamprey scaffold 49 and scaffolds 10 and 27, with a 3-gene minimum per 10- gene window criterion. **c**, The inshore hagfish scaffold FYBX02010521.1, in which the putative *VTR2* is located, is most syntenic with chromosomes of other species containing a *VTR2A or VTR2B* sequence, with a 1-gene minimum per 20-gene window criterion. **d**, Same synteny analyses as in **c**, but with a 2-gene minimum per 20-gene window criterion. **e**, The inshore hagfish scaffold FYBX02010841.1, in which the putative *VTR1* is located, is most syntenic with chromosomes of other species containing a *VTR1A or OTR* sequence, with a 1-gene minimum per 20-gene window criterion. **f**, Same synteny analyses as in **e**, but with a 2-gene minimum per 20-gene window criterion.

To assess whether the interspecies synteny we identified was due to local segmental synteny within a chromosome or to entire chromosomal-scale orthology, we generated dot plots using SynMap2 for entire chromosomes or scaffolds that contained OTR-VTR genes, focusing on comparisons between species that represent major vertebrate lineages and which were sequenced at chromosomal resolution. By examining basal branches, we found the sea lamprey scaffold that contained the combination of *OTR* and *VTR2B* had the highest number of syntenic hits (30–60 genes) to the chromosome in all other vertebrates that had the combination of *OTR* and *VTR2B* (Fig. 3a). We found a similar result between species in chromosomes containing the combination of *VTR1A* and *VTR2A* (Fig. 3b). Exceptions included some fish, in which another chromosome had a similar number of syntenic hits (Fig. 3a, b)—consistent with an extra chromosome paralogue from an additional whole-genome duplication. Mammals were also an exception: here, the orthology of the sea lamprey scaffold containing the *VTR1A-VTR2A* combination was split between two chromosomes (for example, human chromosomes 7 and 12) (Fig. 3b), consistent with a fission event. These results indicate that these two gene combinations are syntenic, because each belongs to a chromosome orthologue of vertebrates after the split with lampreys. The second highest gene hits in most species were to the chromosome that contained the other receptor combination (Fig. 3a, b), which indicates that the chromosomes containing the *VTR1A-VTR2A* and *OTR-VTR2B* combinations may be paralogues from a whole-genome duplication. The third highest number of syntenic gene hits were to chromosomes that contained *VTR1B* or *VTR2C* (in no particular order) (Fig. 3a, b), which suggests possible paralogous segmental duplications. Similar—but not as strong—results were found for an apparent duplicate sea lamprey scaffold that contained one VTR gene (Extended Data Fig. 4a, b, Supplementary Note 1c).

When we used the two scaffolds that contained the *VTR1* and *VTR2* hagfish genes as references, we found fewer syntenic gene hits to chromosomes of other species: chromosomes with the *OTR-VTR2B* and *VTR1A-VTR2A* combinations showed the highest number of hits, with no clear preference between them (Extended Data Fig. 4c–f, Supplementary Note 4). These findings further support a deep ancestral paralogy between chromosomes that contain these two receptor combinations, with possible ancestral chromosome representatives in hagfish. Our findings are consistent with previous studies identifying chromosomal paralogues^14,23–26^, and further reveal newly identified candidate chromosomal paralogues in species with genomes that—to our knowledge—have not yet been compared (sea lamprey versus medaka, frog versus medaka and so on) (Fig. 3).

## Rapid radiation of the VTR1 and VTR2 families

**Extended Data Fig. 5:**
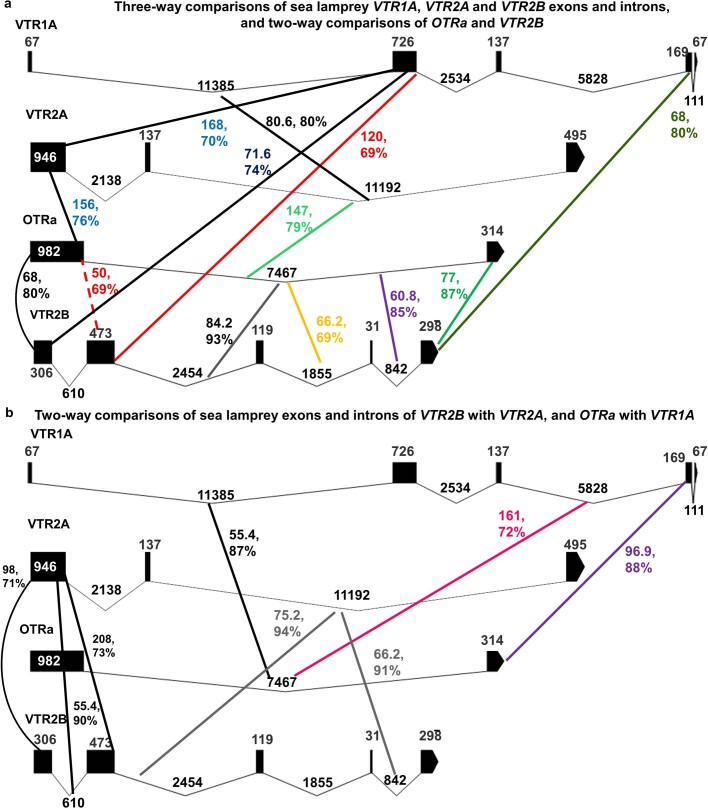
Interspecies BLASTn comparisons between exons and introns of all sea lamprey OTR-VTRs in multiple combinations. **a**, Three-way comparisons of sea lamprey *VTR1A*, *VTR2A* and *VTR2B* exons (boxes) and introns (lines), and two-way comparisons of *OTRa* and *VTR2B*. **b**, Two-way comparisons of sea lamprey exons and introns of *VTR2B* with *VTR2A*, and *OTRa* with *VTR1A*. Maximum scores and per cent identities are shown for the alignments that exceed a threshold (maximum score > 40 and *E* value < 10^−4^). Sequence length is shown in bp.

**Extended Data Fig. 6:**
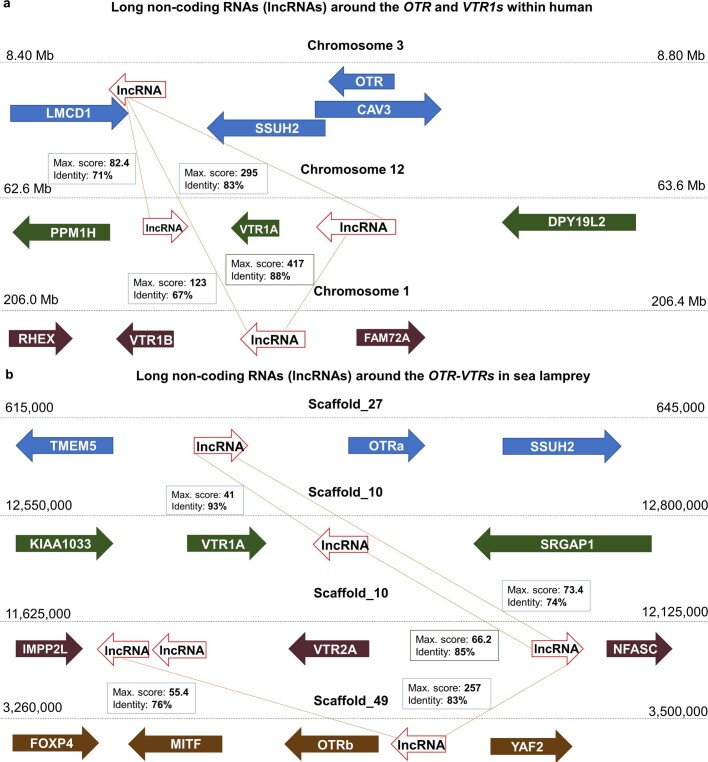
Interspecies non-coding RNA paralogous synteny analyses. **a**, Long non-coding RNAs around the *OTR* and *VTR1* genes within human. **b**, Long non-coding RNAs around the OTR-VTRs in sea lamprey. Lines connect the long non-coding RNAs that shared identity beyond a threshold (maximum score > 40 and *E* value < 10^−4^) in the BLASTn comparisons. Maximum score (bit score) and per cent identity are shown for each pair of long non-coding RNAs. Genomic location is in Mb.

**Extended Data Fig. 7:**
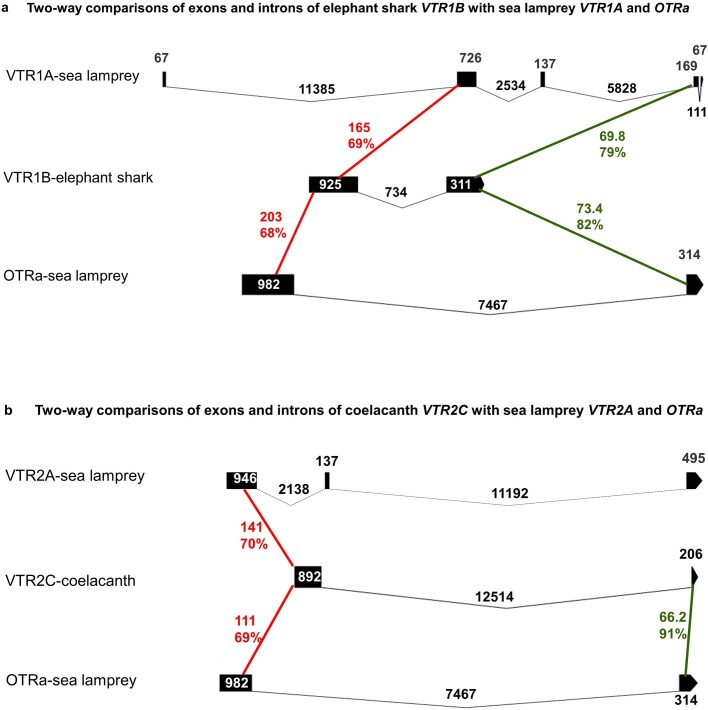
Intraspecies BLASTn comparisons between exons and introns of OTR-VTRs. **a**, Two-way comparisons of exons (boxes) and introns (lines) of elephant shark *VTR1B* with sea lamprey *VTR1A* and *OTRa*. **b**, Two-way comparisons of exons and introns of coelacanth *VTR2C* with sea lamprey *VTR2A* and *OTRa*. Maximum scores and per cent identities are shown for the alignments that yielded results beyond a threshold (maximum score > 40 and *E* value <10^−4^). Sequence length is shown in bp.

**Extended Data Fig. 9:**
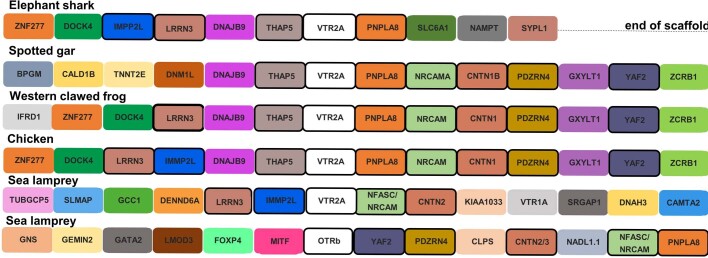
Microsynteny for *VTR2A* across vertebrates and *VTR2A* and *OTRb* within sea lamprey. An approximately 14-gene window around the *VTR2A* orthologue across species is shown. In the sea lamprey, *OTRb* is our revised nomenclature for PMZ_0045207-RA/PMZ_0032217-RA on scaffold 49 (Supplementary Table 14), and *VTR2A* is our revision for PMZ_0042163-RA on scaffold 10 (Supplementary Table 14). Orthologous genes are filled with the same colour; genes found in the territory of the sea lamprey are further outlined in black lines. Further discussion is provided in Supplementary Note 2.

We next assessed paralogues within species to help to determine evolutionary relationships among the receptors. We analysed 10-Mb macrosynteny windows between and within chromosomes of the same species (intraspecies) for all 6 receptors (whether present or deleted). Within species (for example, human), we found paralogous ‘gene families’ in syntenic blocks around all OTR-VTR genes (Fig. 2b, Supplementary Table 9a), which supports the notion that at least parts of these chromosomes are paralogous (human chromosomes 12 and 7, versus chromosome 3). We also found an extra gene-family territory on human chromosome 3 that is syntenic with the *VTR2B* territory (Fig. 2b). However, we did not detect any VTR1 or VTR2 genes within a species that shared substantially more synteny than others (Supplementary Table 9a, b). Further, no gene family was present in the territory of all six present or deleted receptors. At a more microscale level, among the exons and introns of OTR-VTR genes (Extended Data Fig. 5) and in flanking microRNAs and long non-coding RNAs (Extended Data Figs. 6, 7, Supplementary Table 10), we also did not find any one gene with more similarity to another that would allow us to make further conclusions about the evolution of gene subfamilies (for example, sea lamprey and human) (Supplementary Notes 5, 6). Most sequences were too similarly divergent to inform paralogy (Supplementary Note 5). Overall, the lack of further macrosynteny and simple sequence-divergence resolution of paralogues within a species—combined with the better resolution on chromosome orthologues and paralogues between species in which these gene regions reside—suggest a rapid radiation of receptor evolution near the origin of vertebrates.

## Single chromosome origin of OTR-VTRs

To assess receptor origins, we performed ancestral analyses by mapping regions that contain the OTR-VTR genes against reconstructed chromosomes of the vertebrate or chordate ancestor from four independent studies^14,23,25,27^. Chromosome fragments containing the OTR-VTR genes in vertebrates all mapped back to the same reconstructed chromosome (Supplementary Table 11). A previous study^28^ suggested that *VTR2C* maps back to a separate ancestral chromosome: we believe that this is inaccurate because the region that contains the gene we name *VTR2C* is entirely missing from the reconstruction that was used^23^, although it is present in other reconstructions^14,25,27^ that were based on higher-quality amphioxus and sea lamprey genome assemblies (Supplementary Table 11). These findings are consistent with the fact that that vast majority of invertebrates that have been examined have only one *VTR* gene^13,17^ (Supplementary Table 5).

## Synteny, phylogeny and receptor evolution

**Fig. 4:**
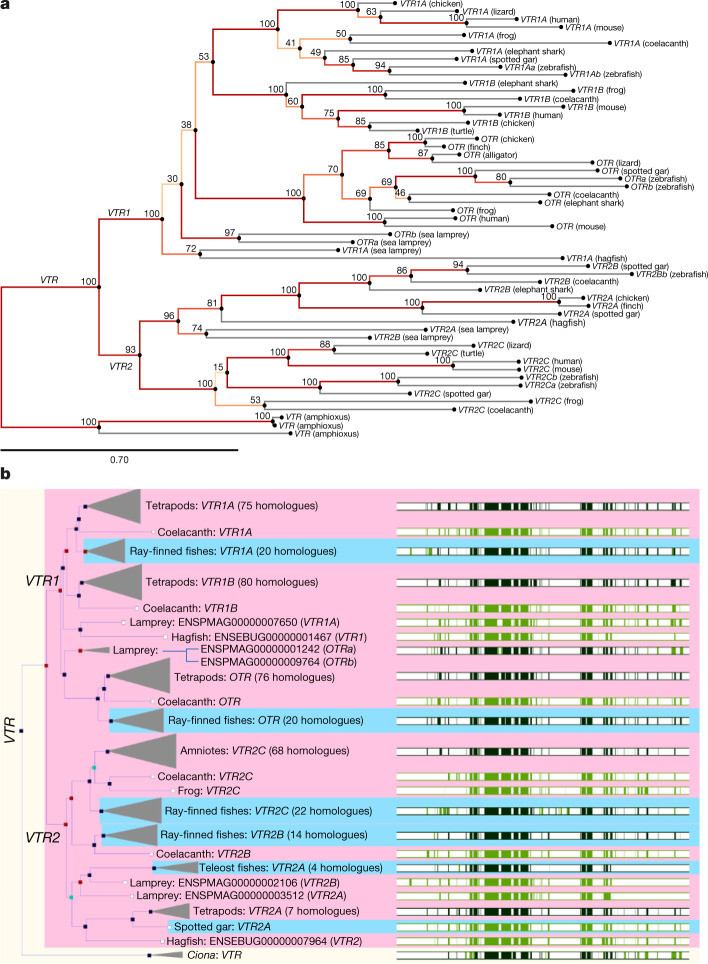
OTR-VTR gene family trees. **a**, Tree topology inferred with the phylogenetic maximum likelihood method on an exon nucleotide alignment (MAFFT), with 1,000 non-parametric bootstrap replicates. Bootstrap values are shown as percentages at the branch points (values <50% were considered less informative). The tree is rooted with the three *VTR* genes we found in amphioxus. The gene names of the current accessions (see Table 1 and Supplementary Tables 4a–e for full list of synonyms) were written over according to our revised synteny-based orthology. Scale bar, phylogenetic distance of 0.78 substitutions. **b**, Tree topology inferred with the phylogenetic TreeFam method on an amino acid alignment generated via the Ensembl ‘gene tree’ tool (gene tree identifier: ENSGT00760000119156). Left, red boxes denote inferred gene duplication node; blue boxes denote inferred speciation events; and turquoise boxes denote ambiguous nodes. Right, green bars denote multiple amino acid alignment made with MUSCLE; white areas denote gaps in the alignment; and dark green bars denote consensus alignments. Gene names are revised according to our synteny-based orthology; Extended Data Fig. 8 shows a tree with the current nomenclature in Ensembl.

We generated phylogenetic trees of the receptor gene family across vertebrates using alignments of both the exon nucleotide (RAXML) and amino acid (TreeFam and TreeBeST5) sequences, and then mapped our revised synteny-based naming onto the tree leaves. BLAST nucleotide comparisons alone, and previous nomenclatures based on these analyses (Table 1), yielded many contradictions with the synteny-defined orthologues (Supplementary Table 12). We believe this is due to BLAST not returning alignments of the entire sequence, which in turn is due to larger lineage divergences between those gene regions (Supplementary Note 5). By contrast, our phylogenetic sequence analyses revealed tree topologies with almost 1:1 consistency to our synteny-defined relationships (Fig. 4).

The combined phylogeny and synteny analyses supported a single *VTR* gene shared with an invertebrate ancestor (that is, represented in sea squirt). This receptor then duplicated into what we designate the ancestral *VTR1* and *VTR2* on the same chromosome (that is, represented in hagfish) (Fig. 4). Thereafter, the trees suggest that these two genes expanded into three genes in the VTR1 subfamily (*VTR1A*, *OTR* and *VTR1B*) and three in the VTR2 subfamily (*VTR2A*, *VTR2B* and *VTR2C*), respectively, with *VTR1A* and *VTR2A* on the same chromosome and the paralogous *OTR* and *VTR2B* on another chromosome. Thereafter, the sister relationship of *VTR1A* and *VTR1B* in both trees suggests that one directly gave rise to the other, after the divergence of jawless fish (based on absence of *VTR1B* in lampreys). Likewise, the sister relationship of *VTR2B* and *VTR2A* in the nucleotide tree is consistent with the synteny findings of one giving rise to the other, and—together—their sister relationship to *VTR2C* is consistent with one of them giving rise to it, after the divergence of sharks (based on absence of *VTR2C* in sharks).

In stark contradiction to the synteny findings, the lamprey *VTR1A* and *OTR* genes each clustered outside of their respective synteny-defined VTR1 homologues among species and the same occurred for lamprey *VTR2A* and *VTR2B* for the VTR2 homologues (Fig. 4), which implies lamprey-specific duplications. One possible explanation for these contradictions is that there could be convergence within the lamprey OTR-VTR genes (possibly owing to higher GC content^29^) or that the divergence was so rapid at the origin of vertebrates that the true relationship is not easy to resolve using gene tree inference. Consistent with the latter, the bootstrap support values are low (72–74%) for a more recent gene duplication. Consistent with the former, the lamprey exon sequences of all 4 receptors were among the highest in GC content (60–69%) compared to other species (Supplementary Table 13). The three amphioxus VTR sequences in our exonic tree cluster within species at 100% support (Fig. 4a), consistent with lineage-specific duplications (Supplementary Note 2b). There were some differences in local relationships in the exon and amino acid trees (Fig. 4a, b), but these did not affect the major conclusions here (Supplementary Note 7).

## A universal nomenclature for OTR-VTR genes

On the basis of the above findings, we propose a universal nomenclature for the OTR-VTR genes in which their root terms follow the ligand names (oxytocin receptor (OTR) and vasotocin receptor (VTR)) and their enumeration terms (1A, 1B, 2A, 2B and 2C) follow their evolutionary history: the numbers 1 and 2 designate the original duplication, and the letters A, B and C designate the subsequent subfamily duplications. The only exceptions we made were the decisions not to rename *OTR* as *VTR1B* or *VTR1B* as *VTR1C* (as the evolutionary history warrants), because we felt this might be too radical of a departure from the common use. This is further justified in that—although there is crosstalk in OT and VT binding to these receptors—OT is the dominant ligand for OTR^30^. For the VTR2 genes, we reordered the enumerations according to the inferred chronological order of duplications: *VTR2A* and *VTR2B* for the genes we found in lampreys, and *VTR2C* for the gene that evolved in the ancestor of bony fishes. This universal nomenclature gives a single name to each gene across vertebrates. The gene that is commonly known as arginine vasopressin receptor 1A (*AVRP1A*) in mammals, vasotocin receptor 4 (*VT4*) in birds and vasotocin receptor (*VasR*) in frogs would, in our revised nomenclature, be called vasotocin receptor 1A (*VTR1A*) (Table 1). The gene that is commonly known as oxytocin receptor (*OXTR*) in mammals, vasotocin receptor 3 (*VT3*) or mesotocin receptor (*MTR*) in birds and frogs, and isotocin receptor (*ITR*) in fish would be called oxytocin receptor (*OTR*). Similar changes from multiple names to a single name apply to the other four receptors (Table 1).

## Interpretations and evolutionary hypotheses

**Fig. 5:**
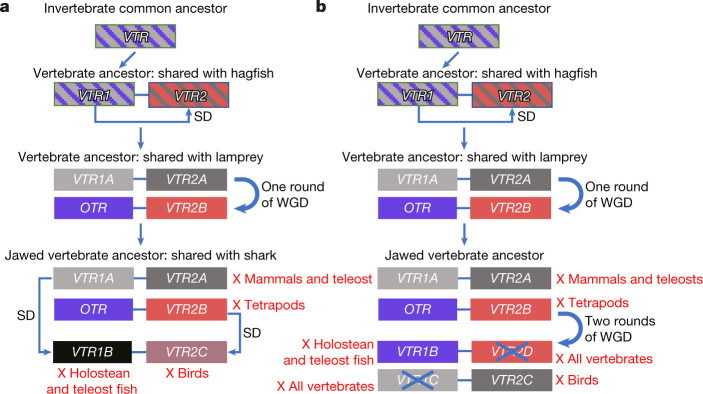
Two proposed hypotheses for the evolution of OTR-VTR genes. **a**, Hypothesis 1 proposes the receptors evolved by an initial segmental duplication (SD), then a one round of whole-genome duplication (WGD), followed by two segmental duplications in different vertebrate lineages and then by losses (red X) in specific lineages. **b**, Hypothesis 2 proposes the initial segmental duplication was followed by two rounds of whole-genome duplication and specific losses (X), including in all vertebrates (blue X). Lines connecting genes indicate that they are on the same chromosome in most species. Alignments between sets of genes indicate the closest related paralogues.

We considered a model of OTR-VTR evolution in the context of two competing hypotheses of vertebrate genome evolution: one round of whole-genome duplication and segmental duplications (Fig. 5a) versus two rounds of whole-genome duplication (Fig. 5b). For both hypotheses, we propose that the single *VTR* in the vertebrate ancestor had a tandem segmental duplication on the same chromosome at over 550 million years ago^31^ that gave rise to the ancestral *VTR1* and *VTR2* genes. Thereafter, in a one round of whole-genome duplication in a gnathostome ancestor, one copy of each of the *VTR1* and *VTR2* genes gave rise to the *VTR1A*-*VTR2A* combination on one chromosome paralogue and the *OTR*-*VTR2B* combination on the other chromosome paralogue. From here, the two hypotheses diverge. In hypothesis 1 (Fig. 5a), a segmental translocated duplication of the chromosomal region containing *VTR1A* gives rise to *VTR1B* in the ancestor of jawed vertebrates at over 500 million years ago and a segmental translocated duplication of the region containing *VTR2B* gives rise to *VTR2C* in the common ancestor of other vertebrates with bony fish at over 450 million years ago. Segmental duplications have been found in other gene families at these evolutionary time points^32,33^. In hypothesis 2 (Fig. 5b), two rounds of whole-genome duplication before the divergence of gnathostomes from cyclostomes lead to four more receptors in the ancestor of jawed vertebrates at over 500 million years ago. Both hypotheses agree with lineage-specific losses of *VTR2B* in the ancestor of tetrapods, of *VTR2A* independently in mammals and teleost fish, of *VTR1B* in holostean and teleost fish, and of *VTR2C* in birds. However, for hypothesis 2 to be true, complete independent losses of thus-far unidentified *VTR1C* and *VTR2D* genes and associated chromosome segments in an extinct species before its divergence into all other vertebrate lineages would be required. Our results are more parsimoniously explained by hypothesis 1 (one-round of whole-genome duplication)^14^ with prior and subsequent segmental duplications (Supplementary Note 8). Such a vertebrate evolutionary scenario is consistent with expectations given a simple random mutational model^34^ that requires as few as 6 mutational steps, whereas models that invoke two rounds of whole-genome duplication require at least 9 steps in our case (Supplementary Note 8) or 12–18 under previous assumptions^34^. Our findings of only two receptor genes (*VTR1* and *VTR2*) in inshore hagfish is more consistent with the paraphyly (separate phyla) of lampreys and hagfishes than the monophyly hypothesis^35^, as it does not require further inference of independent gene losses or gains or incomplete lineage sorting (Fig. 1a). Thus, our findings may have repercussions on a wider and highly debated topic—that of the evolution of vertebrate genomes (Supplementary Note 9).

**Extended Data Fig. 10:**
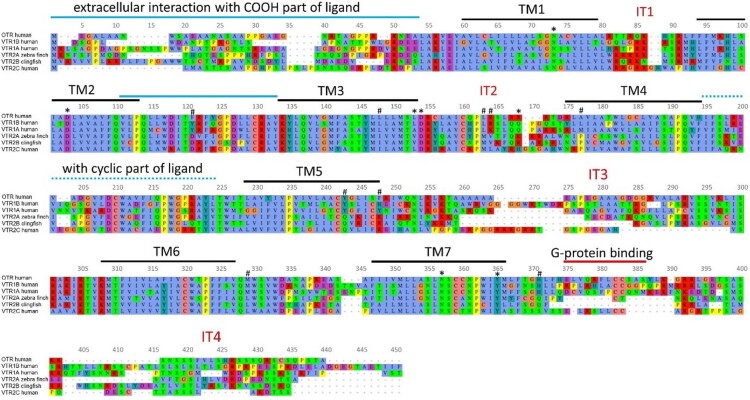
MAFFT alignment of the OTR-VTRs of the best-quality assemblies available (human for *OTR*, *VTR1A*, *VTR1B* and *VTR2C*; zebra finch for *VTR2A;* and clingfish for *VTR2B*). The MAFFT alignment using the FFT-NS-I parameter was visualized with the MSA viewer^46^. The identifiers and protein sequences used, along with the alignment file can be found in https://github.com/constantinatheo/otvt. The functional annotation of transmembrane domains (TM) and intracellular loops (IT) and binding domains is based on findings with *OTR*^47^. Amino acids marked with an asterisk are the OT polar-interacting sites to the receptor; amino acids marked with a # are differences between the VTR1 and VTR2 subfamilies. Colour coding of the amino acids is according to Clustal X (blue, hydrophobic; red, positive charge; green, polar; pink, cysteines; orange, glycines; yellow, prolines; cyan, aromatic; http://www.jalview.org/help/html/colourSchemes/clustal.html).

Our revised understanding of the receptor relationships allows a more holistic view of their functions. We generated a multiprotein coding sequence alignment among the highest-quality assemblies of all six receptors, and found that the seven transmembrane domains and associated polar amino acids that interact with OT or VT have remained highly conserved in sequence or amino acid type, even 550 million years after their common origin (Extended Data Fig. 10, Supplementary Note 10). By contrast, the extracellular OT or VT binding domains and the intracellular G-protein binding domain became highly diversified from one receptor to another, predicting greater diversity in initial ligand binding and subsequent intracellular signalling. Nine amino acid sites distinguish the VTR1 and VTR2 subfamilies (most in or near the transmembrane regions), but only one of these is in the G-protein binding region (Extended Data Fig. 10). All of the receptors use diacylglycerol, inositol triphosphate and Ca^2+^ for second-messenger intracellular signalling—except for VTR2C, which uses cAMP (Supplementary Table 31). The tissues in which the receptors have their highest expression include the brain (except VTR2A), with expression being highest in the adrenal gland. We could not find data available for signalling or expression for VTR2B in fish, but predict it will be similar to members of the VTR2A and VTR2C subfamilies. Finally, our findings that the OTR (represented by lamprey divergence) evolved millions of years before the OT ligand (represented by elephant shark divergence) suggest that the ancestral VT may have originally acted through the OTR before OT evolved. This suggestion is supported by findings that, in some species, OT and VT bind to the OTR at similar efficiencies^8^; a greater response of OTR to OT over VT is found for the first time in teleost fish^8^.

In summary, we believe that our revised evolution-based and universal nomenclature will make translating findings across vertebrates much easier. It will help to inform our understanding of crosstalk between some of the ligands and receptors, our understanding of genome evolution and could serve as a model for a broader universal gene nomenclature.

## Methods

No statistical methods were used to predetermine sample size. The experiments were not randomized, and investigators were not blinded to allocation during experiments and outcome assessment.

### Overall synteny and BLASTn analyses

To define orthology in the *OT*, *VT* and OTR-VTRs in all vertebrates, we used interspecies synteny analyses at three scales: a manual 10-gene window microsyteny analyses using BLAT and BLAST^38,39^ searches and cross-species genome alignments; a more automated 100-gene macrosynteny window using SynFind and GeVo^22^; and automated chromosomal-scale alignments with syntenic dot plots using SynMap2^40^. To define paralogy and further trace the evolutionary history of the genes, we used intraspecies synteny analysis, searching for paralogous genes in 10-Mb windows. Microsynteny was useful for determining orthologous and paralogous relationships between genes in the majority of the vertebrate lineages. Macrosynteny was useful for determining orthologous and paralogous relationships in cases in which the microsynteny was weaker, such as between genes found in lampreys or hagfish with the rest of the vertebrates. Sequence identity was determined using BLASTn to understand relationships between the per cent identity and synteny (Supplementary Table 12). Only results with a bit score >40 and hits with high probability *E* value <10^−4^ were kept. We describe the specific methods for each synteny approach in ‘Microsynteny between species in approximately 10-gene windows’, ‘Macrosynteny between species in approximately 100-gene windows’ and ‘Chromosome-scale macrosynteny between species’.

### Microsynteny between species in approximately 10-gene windows

We ran microsynteny analysis by manually scanning annotated alignments for 5 protein-coding genes before and after each focus gene (Supplementary Table 4a–e) in 35 species spanning all major vertebrate lineages (Supplementary Table 2). The candidate genes in each species (accession number or gene identifier in Supplementary Table 4a–e) were first selected by BLAT and BLAST searches using the UCSC genome browser and alignment (http://genome.ucsc.edu/)^38^ and the SynFind tool from the CoGe comparative genomics research platform^22^. The NCBI and Ensembl^41^ (v.95) database genome alignments were used to identify the neighbouring genes. For the neighbouring genes, we kept in our Supplementary Tables the family gene names used in the genome annotation of each species, even though in some cases—we believe erroneously—different family names have been given to the orthologous gene in different species (for example, *FABP1A* in spotted gar and *FABP1B.1* in stickleback; Supplementary Table 4a). For the species that had more lineage-specific duplications, we labelled the gene that shared more synteny with the orthologue in other vertebrate lineages with ‘a’ (for example, *OTRa*), and labelled the copy with ‘b’ (for example, *OTRb*). We listed the aliases in NCBI and Ensembl for each focus gene in each organism (‘Aliases’ column in Supplementary Table 4a–e) and included the most frequent ones in Table 1. When our target genes appeared to be lost in a species (no initial BLAST hit), we searched the surrounding gene territory to determine whether only the gene of interest or a larger block of genes were deleted, or whether the deletion was due to an incomplete genome assembly or assembly artefact.

For some species with more-fragmented genome assemblies or annotations or greater divergences in NCBI and Ensembl, we analysed other higher-quality assemblies and annotations. This included the VGP zebra finch, Anna’s hummingbird, pale spear-nosed bat and platypus genome assemblies^3^. For the Japanese lamprey, we included previously published synteny data^10^. For the sea lamprey, we used the assembly of the germline genome^14^ and analysed it with BLAST, Genome Browser and Gene Search tools (https://genomes.stowers.org/organism/Petromyzon/marinus). For amphioxus, we used the BLAST and Gene Browser tools available at https://genome.jgi.doe.gov/Brafl1/Brafl1.home.html with the latest version of the amphioxus genome (*Branchiostoma floridae* v.2.0), whereas previously reported data^13^ are based on the first version of the genome (*B. floridae* v.1.0). For the inshore hagfish genome assembly, the contigs were relatively short and not fully annotated, and thus we first BLAT-searched all the *OT*, *VT* and OTR-VTR sequences of all the aforementioned species against the hagfish genome in Ensembl, found two putative OTR-VTRs in two separate contigs in the hagfish assembly, and then used the ‘Region comparison’ tool of Ensembl to map each gene of these contigs against the human, zebrafish and lamprey genomes (Supplementary Table 4f, g). BLAST gave many gene hits in the hagfish genome, but only with short segments aligning to *OT* and *VT* orthologues in other species. Thus, to determine whether they were real *OT* or *VT* orthologues, we used the ‘Gene Tree’ tool of Ensembl that constructs a phylogeny using the entire protein sequence, with the sea lamprey *VT* as reference. For the receptors, we used our data from the SynMap2 dot plots (described in ‘Chromosome-scale macrosynteny between species’) and included in the synteny of the hagfish receptors the gene hits that appear on the chromosomes in which the OTR-VTRs are located in human, chicken, zebrafish and sea lamprey (Supplementary Table 4f, g).

### Macrosynteny between species in approximately 100-gene windows

We generated gene sequence alignments between pairs of species using SynFind^22^ (density, LastZ defaults). This results in a matrix containing syntenic gene-hit values in the reference species relative to query species along with their chromosomal locations. This data matrix was parsed and visualized using a custom R script (https://github.com/ggedman/OT_VT_synteny). First, a 100-gene window centred around a given receptor gene in the reference organism (*x* axis) was defined using biomaRt (v.3.10). As we move 5′ (left) or 3′ (right) from zero (the focus gene) and if synteny exists, the number of gene hits for a given receptor in the query species shows a cumulative increase. This allowed us to identify large stretches of homologous sequences interspersed by divergent sequences.

### Chromosome-scale macrosynteny between species

We used SynMap2^40^ to generate syntenic dot plots of chromosome sequence alignments between species that contain OTR-VTRs (Supplementary Tables 15–30). SynMap2 identifies collinear sets of genes or regions of sequence similarity to infer synteny between two sequences, and generates a dot plot of the results. We used the default parameters (as of December 2018), except for ‘Minimum number of aligned pairs’. This parameter defines the minimum number of homologous genes (based on last default parameters) that should be found in a 20-gene distance for these genes to be considered syntenic and to appear on the dot plot. In more closely related lineages, we selected three as a minimum number (for example, between sea lamprey on the one hand, and Japanese medaka, or zebrafish, frog and chicken genomes on the other); for more distantly related species, we used two (for example, between hagfish on the one hand, and sea lamprey, or Japanese medaka, zebrafish, frog, chicken and human genomes on the other). Additionally, because the hagfish contigs were shorter than most other assemblies (making synteny more difficult to identify), we also ran a dot plot with 1 as the minimum number to search for all possible homologous hits, regardless of synteny.

To test for significant differences, we ran a *χ*^2^ test with distinct samples of genes on the difference of the proportions between the first two chromosomes with the highest number of gene hits, using the number of genes in the super-scaffold of the reference species (for example, sea lamprey) as sample size: Supplementary Table 30 provides confidence intervals, degrees of freedom and *P* values. For cases that reached significance, to confirm that the number of hits between two species was independent of the number of protein-coding genes located on the chromosome of the query organism, we applied a gene density-normalization test to rule out the possibility that the chromosomes with most gene hits were owing to them containing the most genes: we did not find such correlations with our macrosynteny analyses.

### Macrosynteny within species in approximately 10-Mb windows

We primarily used the human genome, as it is the best assembled genome and therefore subject to generating fewer errors. We listed all genes found in a 10-Mb window from the present OTR-VTRs (for example, *OTR, VTR1A*, *VTR1B* and *VTR2C* in mammals) as well as absent ones (for example, *VTR2A* and *VTR2B*, which are absent in mammals). We chose a 10-Mb window because this genomic region size often captured macrosynteny of >40 genes, allowing within- and between-species macrosynteny analyses described in ‘Macrosynteny between species in approximately 100-gene windows’ to be comparable. We then searched each gene in the HUGO Gene Nomenclature Committee Database (https://www.genenames.org/) to classify its gene-family. For the deleted genes, we defined their territories by manually identifying in the human genome the genes around spotted gar *VTR2B* and chicken *VTR2A*; some of these syntenies around the deleted OTR-VTRs had previously been identified^8,9^, which we confirmed.

### Evolutionary history analyses of *OT* and *VT*

We noted annotated DNA transposable elements in the UCSC Genome Browser in close vicinity of the *OT* and *VT* genes (except for the elephant shark genome, which was not annotated for DNA transposable elements), and thus we quantitatively searched for adjacent transposable elements in the human and chimpanzee genomes using RepeatMasker (http://genome.ucsc.edu/)^38^ and obtained information for each specific transposable element using Dfam 2.0^12^. We calculated GC content using ENDMEMO (http://www.endmemo.com/bio/gc.php/). We aligned the introns of human *OT* and *VT* in all possible combinations using DIALIGN^42^ and compared intron lengths using Serial Cloner v.2.6 (http://serialbasics.free.fr/Serial_Cloner.html). For relative *OT* and *VT* orientations, we examined whether they were in the same direction (tail-to-head) or in opposite directions (tail-to-tail) in the annotated positions in each species. In the cases in which *OT* and *VT* were found in opposite directions, we determined which gene was inverted according to the orientation of other genes in the territory. In addition to the genomes used for all other analyses of this study (Supplementary Table 2), we also used the koala (*Phascolarctos cinereus*) (phaCin_unsw_v4.1; GCF_002099425.1) and the grey short-tailed opossum (*Monodelphis domestica*) (MonDom5**;** GCF_000002295.2) genomes to include orientation data from the marsupial clade in Supplementary Table 8.

### Evolutionary history analyses of OTR-VTRs

To assess in which ancestral vertebrate chromosomes the OTR-VTRs originated, we used four ancestral chromosome models from the literature^14,23,25,27^, in which the reconstructed chromosomes were based on different species and different genome qualities. Specifically, human, mouse, dog, chicken and tetraodon genomes were used in ref. ^23^; human, chicken, stickleback, pufferfish, sea squirt, amphioxus, sea urchin, fruitfly and sea anemone genomes in ref. ^27^; human, chicken and sea lamprey (somatic) genomes in ref. ^25^; and chicken, spotted gar and sea lamprey (germline) genomes in ref. ^14^. We searched for the presence of annotated OTR-VTRs in four outgroup invertebrate lineages (through literature review, BLAST and BLAT searches)—namely in sea squirt, roundworm, California sea hare and amphioxus. For the amphioxus genome (*B. floridae* v.2.0), we performed BLAT queries on OTR-VTR FASTA sequences from all species studied using the JGI genome browser (https://genome.jgi.doe.gov/portal/).

To test which sea lamprey receptor(s) most probably represents the orthologous ancestral gene(s), we compared the sea lamprey OTR-VTRs in all possible combinations to each other using BLASTn (same parameters). We compared the exons and introns of the identified genes separately to understand the divergence of the paralogous genes, following a previously proposed paradigm^43^, using the maximum score and per cent identities of the comparisons that were above the threshold (maximum score >40 and *E* value < 10^−4^). We performed a similar analysis for *VTR1B* and *VTR2A* in elephant shark and coelacanth, to test whether sequence identity can help to solve ancestry questions. To shed light on the orthology between the inshore hagfish and the sea lamprey OTR-VTRs, we compared their exons and introns as well.

To analyse conserved non-coding RNA synteny around the OTR-VTRs, we looked for them in alignments in all the species studied in Ensembl, in the miRbase (http://www.mirbase.org/; release 22), and the miRviewer^44^ database (28 February 2012 update). We aligned (BLASTn) long non-coding RNA regions within species (sea lamprey and human).

### Gene tree phylogeny analyses

#### Exonic nucleotide tree

Exonic sequences from all the OTR-VTRs from representative species that had the most-complete assembled genes were aligned with MAFFT under the E-INS-i parameter set, which is optimized for sequences with multiple conserved domains and long gaps. Any incomplete non-lamprey OTR-VTR of less than 1,000 bp was excluded, as alignments on short sequences often lack power to resolve species relationships, resulting in weakly supported gene trees. Because of the basal phylogenetic position of the lamprey, all lamprey OTR-VTRs (754 bp and longer) were included. From this alignment, we generated a phylogenetic maximum likelihood tree using GTRGAMMA model of RAXML (version 8.2.10)^45^, with 1,000 replicates. We calculated the GC content of all the exonic sequences using http://www.endmemo.com/bio/gc.php (Supplementary Table 13).

#### Protein amino acid tree

A maximum likelihood phylogenetic tree was constructed on one representative amino acid sequence for every gene in every species, using TreeFAM and TreeBeST5 pipeline in the gene tree tool package of Ensembl (https://www.ensembl.org/info/genome/compara/homology_method.html). Thereafter, we manually curated the Ensembl tree (gene tree identifier: ENSGT00760000119156) using the universal nomenclature that we propose here. All the sequences used to generate both trees, sequence alignments and Newick files can be found at https://github.com/constantinatheo/otvt.

### Reporting summary

Further information on research design is available in the Nature Research Reporting Summary linked to this paper.

## Online content

Any methods, additional references, Nature Research reporting summaries, source data, extended data, supplementary information, acknowledgements, peer review information; details of author contributions and competing interests; and statements of data and code availability are available at 10.1038/s41586-020-03040-7.

## Supplementary information

Supplementary Information This file contains Supplementary Notes 1-10, Supplementary Tables 1 and 31, and Supplementary Figures 1-7.

Reporting Summary

Supplementary Tables This file contains Supplementary Tables 2-30. (XLSX)

## Figures and Tables

**Extended Data Fig. 8:**
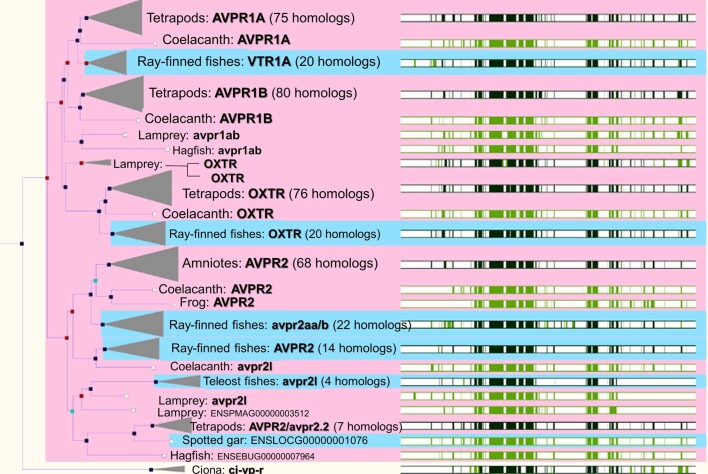
Protein phylogenetic tree for OTR-VTRs with the currently used gene nomenclature. The same amino acid tree as in Fig. 4b, but labelled with the nomenclature used to date. Further variations within large vertebrate groups, such as tetrapods (for example, *VT1* to *VT4* in birds, *AVPR3* in mammals and *AVPR4* in fish), are not shown.

## Data Availability

All the data used in this study can be found in Supplementary Tables 2–30, and at https://github.com/constantinatheo/otvt. Any other relevant data are available from the corresponding authors upon request.

## Code availability

The code used in this study can be found at https://github.com/constantinatheo/otvt and https://github.com/ggedman/OT_VT_synteny.
