# Supplementary material for: Universal nomenclature for oxytocin–vasotocin ligand and receptor families
Source: Nature. 2021 Apr 28;592(7856):747–55. doi: 10.1038/s41586-020-03040-7 (PMC8081664; doi:10.1038/s41586-020-03040-7)
Supplement: Supplementary file 1 — This file contains Supplementary Notes 1-10, Supplementary Tables 1 and 31, and Supplementary Figures 1-7. [file 41586_2020_3040_MOESM1_ESM.pdf]

nature research
https://doi.org/10.1038/s41586-020-03040-7

Supplementary information

Universal nomenclature for oxytocin–vasotocin ligand and receptor families

In the format provided by the authors and unedited

Universal nomenclature for oxytocin-vasotocin ligand and receptor families

Constantina Theofanopoulou*[1,2,3], Gregory Gedman[1], James A. Cahill[1], Cedric Boeckx[2,3,4] and Erich D. Jarvis*[1,5]

[1]Laboratory of Neurogenetics of Language, Rockefeller University, New York, USA
[2]Section of General Linguistics, University of Barcelona, Barcelona, Spain
[3]University de Barcelona Institute for Complex Systems, Barcelona, Spain
[4]ICREA, Passeig, Lluis Companys, Barcelona, Spain
[5]Howard Hughes Medical Institute, Chevy Chase, Maryland, USA

* corresponding author emails: ktheofanop@rockefeller.edu and ejarvis@rockefeller.edu

Supplementary Information

Supplementary Notes

Supplementary Note 1: Oxytocin and vasotocin processing and functions

Both oxytocin (*OT*) and vasotocin (*VT*) are derived from precursor molecules. In the brain, these precursors are processed during the axonal transport from the synthesizing source, the hypothalamus, to other locations[6]. The other locations include the pituitary, which releases oxytocin or vasotocin into the blood system, where they act as hormones. In other parts of the brain, they are released synaptically in response to neural activity, onto neurons containing their receptors, acting as neurotransmitters (see review[46] and references therein).

For the oxytocin gene structure, three exons give rise to a prepropeptide: the first exon encodes the oxytocin hormone, the tripeptide processing signal (GKR), and the NH2-terminal residues of neurophysin I; the second exon encodes the central part of neurophysin I; and the third exon encodes the COOH-terminal region of neurophysin II. The OT prohormone undergoes cleavage and other modifications while it is carried down the axon to terminals located in the posterior pituitary[47,48]. The products of these processes, OT and neurophysin I, are stored within neurosecretory granules until their release is elicited[49].

For vasotocin, the three exons also give rise to a prepropeptide: the first exon encodes a signal peptide, the vasotocin hormone, and the NH2-terminal region of neurophysin II; the second exon encodes the central region of neurophysin II; and the third exon encodes the COOH-terminal region of neurophysin II and the glycopeptide copeptin[50]. Once this prepropeptide complex of vasotocin, copeptin and neurophysin II is synthesized, it is packaged in granules for axonal transport to the posterior pituitary[48].

Oxytocin and vasotocin have diverse functions, some of them conserved across some lineages. These include oxytocin's involvement in sexual behaviors in mammals, birds, amphibians and invertebrates, or vasotocin's involvement in antidiuresis in mammals, birds, reptiles, fish and invertebrates (Supplementary Table 1). Other functions are possibly unique to a lineage, including oxytocin's role in ossification and sleep in mammals, or vasotocin's role in appetitive response in amphibians. These two ligands show also some overlapping functions (e.g. involvement in maternal, social and sexual behaviors), which is expected, based on their common origin[12]. For a full list of biological functions that the two ligands take part in in vertebrates and invertebrates see Supplementary Table 1.

The diverse functions of oxytocin and vasotocin depend on the synthesis sites in the brain and several peripheral organs, the release sites into the blood or other brain regions, and the *OTR-VTRs* that they bind to at these sites. The *OTR-VTRs* are classical 7-transmembrane G-protein coupled receptors. When the peptides bind to these receptors, they cause a series of signal transduction cascades with either excitatory or inhibitory actions on Ca^{2+} and other messengers and transcription of specific genes. Each receptor will have differences in their function according to differences in their cell types and tissue of expression, and their functional sequences. Some of the expected differences in the context of the new nomenclature of this study are presented in Supplementary Note 10.

Supplementary Note 2: Lineage or species-specific *OT-VT* and *OTR- VTR* duplications.
a, Pale spear-nosed bat OT and VT duplications. In the pale spear-nosed bat genome, a high-quality assembly produced by the VGP and Bat1K projects, we found the *OT* and *VT* region duplicated twice, resulting in three *OT* and three *VT* genes. We consider these duplications real, because single Pacbio reads, gapless contigs, and Bionano optical maps spanned through the entire region, without any noticeable assembly errors. This could represent bats generally, and explain the poor short-read assembly for this region in the megabat assembly with *OT* appearing as an only gene in one scaffold (Supplementary Table 4a; Extended Data Fig. 1b,c).

b, Amphioxus VT and VTR duplications. Amphioxus has 3 *VT* genes, with two of them located on the same scaffold (135) next to each other, and the third on a different scaffold (480) (Supplementary Table 5). This third *VT* seems to have been a part of a larger segmental duplication of at least 4 genes, whose synteny we find unaltered on scaffold 135 (Supplementary Table 5). As far as we are aware, this is the first study to report these amphioxus-specific *VT* duplications, while other studies[12,17] that used an earlier version of the same assembly (v. 1.0) report only one *VT* gene on scaffold 135. Amphioxus has also 3 *VTR* genes, with two of them located on the same scaffold (339), and a third on a separate scaffold (97). As revealed in our exonic tree, these three *VTRs* branch with 100% confidence with each other, which suggests that they are, as well, species-specific duplications or possible false duplications due to not phasing haplotypes.

c, Sea lamprey OTR duplication. We found an additional *OTR-VTR* in sea lamprey that is located on scaffold 49 (Supplementary Table 14). This receptor does not appear as a pair with another *OTR-VTR* on the same scaffold, unlike the other two *OTR-VTRs* combinations in lampreys. In both the exonic and the protein phylogenetic trees, this receptor branches directly with high confidence (97% and 100% bootstrap support, respectively) with the sea lamprey *OTR*-ortholog (Fig. 4a,b). It has the highest hits with the chromosomes that have the *OTR-VTR2B* combination in other vertebrates (Extended Data Fig. 4a). However, it shares synteny with 5 genes in the *VTR2A* territory in the rest of vertebrates, while the sea lamprey-*VTR2A* shares 4 genes with the *VTR2A* region in other vertebrates (Extended Data Fig. 9). In order to resolve this conundrum, we ran an intraspecies SynMap2 analysis between the entire sea lamprey scaffold 49 (containing the duplicated *OTR-VTR*) with sea lamprey scaffolds 10 (where *VTR1A-VTR2A* are located) and 27 (where *OTR-VTR2B* are located). We found that scaffold 49 shares more synteny with scaffold 27 (52 genes) than with scaffold 10 (11 genes) (Extended Data Fig. 4b). This suggests that a large segment from scaffold 49 was most likely duplicated from scaffold 27, including the *OTR* we found there. Pairing this syntenic result with the phylogenetic result, we propose that this receptor is an *OTR*-duplication. We further propose the name *OTRa* for the sea lamprey *OTR*-vertebrate ortholog and *OTRb* for the segmentally duplicated gene (Supplementary Table 14).

In sea lamprey, we also noted that three genes (*PPM1H-MON2-USP15*) that are next to *VTR1A* in other vertebrate species were not found syntenic with sea lamprey *VTR1A* (Supplementary Table 4c) on scaffold 10. Instead, these three genes were on sea lamprey scaffold 22, and there was space in the alignment to human and elephant shark (GRCh38.p13 Chromosome 12: 62,935,701--63,150,942; elephant shark v6.1.3 Scaffold NW_006890068: 1812220-2000126). This is evidence for a translocation event of these genes to *VTR1A*'s territory post-lamprey divergence. Alternatively, this apparent translocation and the duplications on scaffold 49 could also be signs of miss-assemblies that could be corrected with improved assemblies of complex genomes like the lamprey.

d, Teleost fish VTR1 and VTR2 family duplications. We identified 2 copies each of *VTR1A* and *VTR2C* in all the teleost fish we analyzed (stickleback, platyfish, medaka, tilapia and zebrafish); 2 copies of *OTR* in all of them except for the stickleback, who only had the first copy; 2 copies of *VTR2B* in all of them except for the platyfish, who only had the second copy, and zebrafish who only had the first copy; the first copy of the *VTR2A* was found only in stickleback and platyfish, while the second only in zebrafish (Supplementary Tables 4b-4e). We surmised that these additional copies and deletions are due to large-scale genome or chromosome segmental duplications in teleost, followed by some losses. Based on synteny with other vertebrate lineages, we named the copy that had the most synteny to other vertebrates with 'a' and the one with less synteny with 'b', for example *OTRa* and *OTRb* in zebrafish (Supplementary Table 4b).

e, Spotted gar VTR2C duplication. In spotted gar, we found an additional receptor (Ensemble ID: ENSLOCG00000000052; GenBank ID:102698801) which was present as the only gene in a scaffold (Scaffold AHAT01043512.1:3,326-8,973), indicating a fragmented assembly, and so we were not able to retrieve synteny data. But the gene branched directly (100% bootstrap support) with the spotted gar *VTR2C* in the 'Gene Tree' available for this *gene* in Ensembl (ENSGT00950000182665). However, we suspect that this could be a false haplotype duplication assembly error[51], as the region is 99.9% identical to the *VTR2C* gene region.

Supplementary Note 3: SynFind macrosynteny analyses

We performed SynFind[21] analyses on alignments up to 100 gene macrosynteny windows around the receptors. This macrosynteny analysis was consistent with our manual microsynteny analyses. For example, for closely related species, the synteny around the receptors was strongest between pairs of genes we annotated as orthologs, and not between those we annotated as paralogs (e.g. human vs chimpanzee; Extended Data Fig. 3a-d), with an average of 15 out of 20 genes being syntenic. For intermediate relationships, as expected, fewer genes (7-20) in the same window size were syntenic, on one or both sides of the receptor, but still consistent with our orthology designations (e.g. human vs chicken; Extended Data Fig. 3e-g). For the most distant relationships, SynFind was not as sensitive in picking up genes in synteny as our manual analyses, but the genes with the most syntenic hits still conformed to our orthology designations (e.g. human or chicken vs fish or frog species; Extended Data Fig. 3i-o). In cases where our manual analyses noted large segmental deletions (e.g. chicken *VTR2C* region; Extended Data Fig. 3h), or local rearrangements on one side of a receptor (e.g. in fish; Extended Data Fig. 3l-p), we noted absence of synteny for the differential rearranged region.

Supplementary Note 4: SynMap2 findings of *VTR1* and *VTR2* in hagfish

The scaffolds where the *OTR-VTRs* are located according to sequence identity in hagfish did not show significant synteny difference with any scaffold/chromosome in any species (Extended Data Fig. 4c-f; Supplementary Tables 20-29), but this was partly expected because the reads are short and the evolutionary distance is longer. Nevertheless, when these hagfish scaffolds were aligned against the genomes of other vertebrate species, they had the most gene hits in chromosomes where *VTR1A* and *VTR2A*, or *OTR* and *VTR2B* were located. Specifically, hagfish scaffold FYBX02010521.1 containing a *VTR1* gene (ENSEBUG00000001467), whose sequence clusters to *VTR1A* in the protein and exonic trees (Fig. 4a,b), showed most syntenic hits with the zebrafish and the human chromosomes where *VTR1A-VTR2A* are located, and sea lamprey scaffold 27 and the chicken chromosome where *OTR-VTR2B* are located (Extended Data Fig. 4c,d). Hagfish scaffold FYBX02010841.1 containing a *VTR2* gene (ENSEBUG00000007964), whose sequence clusters to *VTR2A* in the protein and nucleotide exonic trees (Fig. 4a,b), showed synteny to only sea lamprey scaffold 10 under strict parameters (Extended Data Fig. 4e), and under less strict parameters most to chromosomes in other species equally where *VTR2A* or *OTR* are located (Extended Data Fig. 4f; see also Supplementary Table 4f,g for more detail). These findings suggest that these two scaffolds in hagfish have a *VTR1* and a *VTR2* gene respectively that may represent ancestral chromosomes that contain the *OTR-VTR2B* and *VTR1A-VTR2A* combinations in other vertebrates.

Supplementary Note 5: Sequence identity alone is insufficient to infer evolutionary history

To obtain further clarity on the homologies between the *OTR-VTRs* sequences, we aligned all *OTR-VTRs* of several species against each other using BLASTn (Supplementary Table 12). Orthologous genes defined by synteny were not always the ones with the highest max scores or identities defined by BLASTn. For example, human *OTR* defined by synteny was returned with highest identity to chicken *OTR* (78%) and frog *VTR1B* (82%), not frog *OTR* (71%). Sea lamprey *OTR* had higher BLASTn-defined identities to the other receptors other than *OTR* in other species (Supplementary Table 12). We also tested whether an intron-exon alignment between putative segmentally duplicated genes (like *VTR1B* and *VTR2A*) against the rest of the receptors would reveal ancestry, but the identities and max scores were too similar to resolve such questions (Extended Data Fig. 7). These findings further illustrate that BLASTn-defined sequence identity alone is not sufficient to infer the evolutionary history of some genes, where synteny and phylogenetic analyses of sequence alignments more clearly reveal them. We believe the main issue is that BLAST searches return results for part of the sequence in the alignment, whereas synteny and phylogenetic RAXML analyses uses the entire sequence. This means that although part of a sequence of two paralogous genes may be more identical than their homologs, the entire sequence of the paralogs are not.

Supplementary Note 6: lncRNA synteny reveals divergences after duplications

Conserved microRNAs and long non-coding RNAs (lncRNAs) have been used to help infer the evolutionary history of adjacent protein coding genes[13]. We searched for microRNAs in the territory of the *OTR-VTRs* and identified two: mir-let-7i and mir-718. Mir-let-7i was found next to *PPM1H* (which, in turn, is next to *VTR1A*), which we found conserved from human to the elephant shark (Supplementary Table 10). The presence of this microRNA brings further evidence for our proposed orthology of *VTR1A* in these species (Supplementary Table 4c). Mir-718 is located some genes away from *VTR2C* and is conserved only in some mammalian species, hence it could not help us resolve the evolutionary history of this gene (http://people.csail.mit.edu/akiezun/microRNAviewer/all_mir-718-align.html).

We also searched for lncRNAs next to *OTR-VTRs* across species, but did not find any conserved ones. This may not be surprising, since lncRNAs evolve rapidly, with >70% of lncRNAs having no sequence-similar orthologs in species separated by >50 million years of evolutionary divergence[52]. Less than 100 lncRNAs have been traced to the last common ancestor of tetrapods and teleost fish[52]. However, we did find lncRNAs adjacent to some *OTR-VTRs* within species. Sequence identities were high (67-93%) between almost all lncRNAs flanking human and lamprey *OTR-VTRs* (Extended Data Fig. 6a,b), rendering it impossible to gain insight on the evolution of these genes. What this suggests instead is that these lncRNAs in lamprey could stem back to a single lncRNA next to the putative *VTR* progenitor gene.

Supplementary Note 7: Differences in exon and amino acid phylogenetic trees
In the exon nucleotide tree (Fig. 4a) the sea lamprey *VTR2A* and *VTR2B*, and the hagfish *VTR2*, clustered as outgroups of the *VTR2A*/*B* clade, while in the protein tree (Fig. 4b) they branched as outgroups of the teleost-*VTR2A* sequences, and the spotted gar and tetrapod-*VTR2A* sequences, respectively. In the nucleotide tree (Fig. 4a), sea lamprey *VTR1A*, *OTRa* and *OTRb* and hagfish *VTR1* clustered as outgroups of the *VTR1A*/*B*/*OTR* clade, whereas in the protein tree (Fig. 4b), sea lamprey *VTR1A* and hagfish putative *VTR1* clustered as an outgroup to *VTR1A*/*B* of other vertebrates, and lamprey *OTRa* and *OTRb* as an outgroup of the *OTR* clade. In both trees, lamprey *OTRa* and *OTRb* clustered together with strong bootstrap support, indicating they are the result of a duplication. These results agree with our exon and intron BLASTn alignments, where we found that the first exon of hagfish putative *VTR2* matched the full sequence region of the first exon of lamprey *VTR2A* (75%), while the second exon of hagfish *VTR1* matched the second exon of lamprey *VTR1A* (67%).

Supplementary Note 8: Differences between our and other published hypotheses
Both our proposed hypothesis of the receptor gene family evolution (Fig. 5) differ from what has been proposed in previous studies[9,10]. Even though Mayasich and Clarke[10] put forward a 1R of WGD plus segmental duplications scenario, they were not able to label each *OTR-VTR* they had identified in the sea lamprey somatic genome as specifically *OTR* or *VTR1A* (translated to terminology of the current study); labels were left ambiguous (e.g. “OXTR/V1A/B”). This is because a 1:1 correspondence of orthologs is very difficult draw at this level of evolution, due to whole-genome and whole-chromosome duplications[13]. Despite these difficulties, we managed to obtain higher resolution of orthology through our microsynteny and SynMap2 analysis at a chromosomal/scaffold level and higher quality genome assemblies. According to this analysis, sea lamprey scaffolds 27 and 10 share significant synteny with other species’ chromosomes where *OTR* with *VTR2B* and *VTR1A* with *VTR2A* reside, respectively.

According to the 2R of WGD scenario, Lagman and colleagues[9] and Mayasich and Clarke[10] proposed that they occurred in the cyclostome ancestor. Mayasich and Clarke[10] specifically note that *VTR1B* and *VTR2C* (terminology of the current study) had evolved already in the cyclostome ancestor and were deleted in the sea lamprey. In our analysis of the germline sea lamprey genome, we did not find evidence for either *VTR1B* or *VTR2C* being lost. Furthermore, different recent analyses have revealed that there was 1R of WGD that most likely occurred in the lamprey/cyclostome ancestor, not 2R[11,35]. So according to our proposal, *VTR1B* and *VTR2C* appeared post lampreys/cyclostomes divergence. Lastly, unlike[10], we do not believe that a lamprey-specific 3R of WGD gave rise to what we designate as *OTRb* on lamprey scaffold 49, because our synteny and phylogenetic data suggest this gene was a segmental duplication of *OTRa* on scaffold 27.

In terms of the number of mutations events for each hypothesis for the receptor family evolution, based on[35] a 1R-WGD scenario would require 6 steps: 1 step for the 1R-WGD; 2 steps for the 2 segmental duplications that gave rise to *VTR1B* and *VTR2C*; 2 steps for the independent fission of the *VTR1A-VTR2A*-containing chromosomes in mammals and teleost fish; and a last step for the fission of the *OTR-VTR2B*-containing chromosomes in tetrapods. A 2R-WGD scenario would require ~9 steps: 2 steps for the 2R-WGD; 2 additional steps for the hypothetical '*VTR1C*' and '*VTR2D*' deletions; preceded or followed by another 2 steps of translocations/fissions/deletions of their larger chromosomal regions; 2 steps for the independent fission of the *VTR1A-VTR2A*-containing chromosomes in mammals and teleost fish; and a last step for the fission of the *OTR-VTR2B*-containing chromosomes in tetrapods.

Supplementary Note 9: Our analysis supports hypothesized fusions and fissions in vertebrate genome evolution

The inclusion of 35 vertebrate genomes in our study was crucial for our understanding of the chromosomal fusions and fissions in vertebrate evolution for the chromosomes where *OTR-VTRs* are located. Our finding that *VTR1A* and *VTR2A* are on the same chromosome/scaffold in all vertebrate species except teleost fish and mammals, concurs with reconstructions of putative ancestral tetrapod chromosomes[25] and of putative pre-teleost duplicated-chromosomes[23], where the chromosomes where *VTR1A* and *VTR2A* are located date back to a single putative ancestral chromosome. This would mean that in the bony fish-ancestor, this chromosome was likely subjected to fissions independently in teleost fish and in mammals. Considering this, we can hypothesize and expect that when the coelacanth and hagfish genomes will be assembled at a chromosome-level, the scaffolds where we find *VTR1A* (or *VTR1)* and *VTR2A* (or *VTR2*) will also belong to the same chromosome. For the fission in mammals, we noted that most species' chromosome break near the lost *VTR2A* is directly adjacent to the centromere of this chromosome (Supplementary Table 4c; Column H), where syntenic *CNTN1* is located. In the same vein, in mammals and birds, the chromosomal break near the lost *VTR2B* is adjacent to the telomere of that respective chromosome (Supplementary Table 4b; Column I), where the syntenic paralogous *CNTN4* and *CNTN6* genes are located. Centromere and telomeres are known hotspots of recombination and other chromosome rearrangements[53,54].

Supplementary Note 10. Proposed distinctions of oxytocin and vasotocin receptors

With a revised nomenclature and more complete understanding of the relationships of the *OTR-VTRs*, it becomes possible to more readily compare differences in their functions, according to sequence differences and known functional studies. Most functional studies have been conducted in rodents and humans, and thus, the two receptors that do not exist in mammals are less characterized. Nevertheless, there is sufficient information to infer functional differences.

To compare the gene expression patterns of the *OTR-VTRs*, we searched the NCBI page of each receptor in human (*OTR, VTR1A, VTR1B, VTR2C*) in the 'Expression' category ('RNA sequencing of total RNA from 20 humans'). For the receptors not present in human, we only found broad gene expression data for chicken *VTR2A* in the EBI Gene expression atlas (https://www.ebi.ac.uk/gxa/home)[55,56], but not for *VTR2B*. The brain contained among the highest levels for 4 of the 5 receptors assessed (*OTR, VTR1B, VTR2A, and VTR2C*; Supplementary Table 31). *VTR1B* was highest in the adrenal gland. There were no consistent differences between the *VTR1* and *VTR2* subfamilies that we could recognize.

In terms of signaling, the activity of all *OTR-VTRs*, except *VTR2C*, is mediated by $G_{q/11}$ proteins which activate a phosphatidylinositol-calcium second messenger system (Ca^{2+}), whereas the activity of *VTR2C* is mediated by G_s proteins that activate adenylate cyclase

(cAMP)[47,57,58]. *OTR* has been shown to also couple to G_i proteins[59] (Supplementary Table 31).

We sought to identify amino acid changes that might underlie these gene expression and signaling differences by comparing MAFFT alignments of the *OTR-VTRs* of the best-quality assemblies available (human for *OTR*, *VTR1A*, *VTR1B*, *VTR2C*; zebra finch for *VTR2A;* and clingfish for *VTR2B*) (Extended Data Fig. 10; functional annotation based on[47]). We found that the NH2-terminal extracellular domain that binds to *OT* and *VT*, and the COOH-terminal domain that has the G-protein coupled binding part of the proteins were the most varied across receptors. This means that binding of *OT* and *VT* on the outside of the cell, and intracellular signaling through different G-proteins on the inside of the cell will likely be the most varied functions across receptors. In contrast, once *OT* or *VT* binds to the NH2-terminal, 6 of the 7 amino acid polar residues (Extended Data Fig. 10; amino acids marked with an '*'), where *OT* or *VT* interact, were conserved across all receptors. The intracellular loops (IT) were less conserved than the extracellular loops, of which the latter interact with *OT* and *VT*. The 7 transmembrane domains (TM1-TM7) were the most conserved either in sequence or amino acid type across the receptor family.

We additionally identified sites that distinguish the *VTR1* from the *VTR2* subfamilies (Extended Data Fig. 10; amino acids marked with an '#'): These included differences in the TM3, TM4, and TM5, with site 177 of the alignment in TM4 being a proline (P) in the *VTR2s*, which would make an important folding difference, since prolines have the largest effect on folding kinetics[60]. Other site differences in the 1st extracellular loop (site 121) and right before the G-protein (site 371) could indicate that there may be a difference in how these two receptor subfamilies bind to *OT* and *VT*, and how they signal inside the cell (Supplementary Table 31; 'Signaling differences').

Overall, the sequence differences we identified in these two subfamilies support our phylogenetic tree findings on two progenitor *VTR1* and *VTR2*, from where *OTR*, *VTR1A*, *VTR1B* and *VTR2A*, *VTR2B*, *VTR2C* expanded, respectively. The differential signaling of only *VTR2C* lends credence to our finding that this gene has been by itself the most recent segmental duplication.

Supplementary Tables

Old nomenclature	Universal Vertebrate Revision	Functions
Oxytocin, Neurophysin, Mesotocin, Isotocin, Glumitocin, Valitocin, Aspargtocin	Oxytocin (*OT*)	**Mammals:** drinking[61], eating[61], female pregnancy[62], grooming[63], heart development,[64] lactation[65], mating[66,67], aggression[68], memory[69], blood pressure regulation[70], ossification[71], uterine contractions[72], digestive system regulation[73], pain perception[74], estradiol response[75], sleep[26], social behavior[76], sperm ejaculation[77], sensory perception[78]<br><br>**Birds:** pair bonding[79], social behavior[80], locomotion[81], food intake[81], aggression[82]<br><br>**Reptiles:** nesting behavior[83], egg-laying[83]

		**Amphibians:** reproductive behavior[84]<br><br>**Coelacanths: -**<br><br>**Fish:** social vocalizations[85,86], social behavior[87], nocifensive behavior[88]<br><br>**Sharks:** probably osmoregulation (based on gene expression in kidney, rectal gland and intestine)[12]<br><br>**Lampreys/Hagfishes: -**
Arginine Vasopressin,<br>Neurophysin II,<br>Lysine vasopressin,<br>Phenypressin,<br>Vasotocin	Vasotocin (*VT*)	**Mammals:** apoptosis regulation[92], locomotion[93], maternal behavior[94], grooming[94], arterial blood pressure regulation[95], vasoconstriction regulation[96], antidiuresis[97], thermoregulation[98], social behavior[99], memory[100], pair-bonding[101]<br><br>**Birds:** antidiuresis[102], sexual behavior[103], singing[104,105], social behavior[106]<br><br>**Reptiles:** antidiuresis[107], social rank[108], nesting[109], oviposition[110], parturition[111]<br><br>**Amphibians:** vocalizations[112], egg-laying[113], sexual behaviour[114], appetitive response[115]<br><br>**Coelacanths: -**<br><br>**Fish:** courtship[116], aggression[117], vocalization[86], social behaviour[118], seasonal changes[119], circadian rhythm[120], blood pressure[121], antidiuresis[122]<br><br>**Sharks:** probably ovulation and parturition (based on gene expression in the ovary)[12]<br><br>**Lampreys/Hagfishes:** pherormone release regulation[123], carbohydrate metabolism[124]<br><br>**Invertebrates:** diuretic signaling pathway[125,126], osmoregulation[89], egg-laying[89], long-term memory[90], reproduction[91], carbohydrate metabolism[91]

Supplementary Table 1: Main biological functions of *OT* and *VT* genes in vertebrates and *VT* homolog in invertebrates. First column: old nomenclature for *OT* and *VT* genes in different lineages. Second column: our revised universal vertebrate nomenclature. Third column: major biological

functions of *OT* and *VT* in each lineage. For the mammalian functions, we reviewed the processes included in the ‘Gene ontology’ category of the *OT* in humans and rodents. For the remaining lineages, we performed a Pubmed and Google Scholar literature review. For invertebrates, we have added all the functions we found for the plausible *VT* homolog. Color shading, terms that fall under the same general biological function (e.g. purple for ‘sexual behavior’ processes: courtship, pair-bonding, grooming, sperm ejaculation, reproductive behavior, sexual behavior etc.; light green for ‘mothering’ processes: female pregnancy, uterine contractions, egg-laying, nesting etc).

For Supplementary Tables 2-30, see ‘Supplementary Tables 2-30_Theofanopoulou et al.’ excel file.

Receptor	Organism Characterized	Expression Differences	Signaling Differences
VTR1A	Vertebrates	adrenal, thyroid, uterus, liver	$G_{q/11}$ proteins<br>DAG/IP3/Ca^{2+}
OTR	Vertebrates	skeletal muscle, brain, prostate, trachea, uterus	$G_{q/11}$/G_i proteins<br>DAG/IP3/Ca^{2+}
VTR1B	Mammals/Birds	kidney, small intestine, uterus, brain	$G_{q/11}$ proteins<br>DAG/IP3/Ca^{2+}
VTR2A	Birds/Reptiles	brain, colon, testis	$G_{q/11}$ proteins<br>DAG/IP3/Ca^{2+}
VTR2B	Fish	no data available	no data available
VTR2C	Mammals/Fish	brain, thymus, heart, lung	G_s proteins<br>cAMP

Supplementary Table 31: Gene expression and signaling differences of *OTR-VTRs* in the organisms where they have been characterized. For gene expression patterns, data on the *OTR, VTR1A, VTR1B* and *VTR2C* come from each gene’s NCBI page on human (‘Expression’ category -‘RNA sequencing of total RNA from 20 humans’); on the *VTR2A* from the EBI Gene expression atlas (https://www.ebi.ac.uk/gxa/home)[55,56]. We list the four top tissues where each receptor had the highest gene expression levels among all the tissues tested.

Supplementary Figures

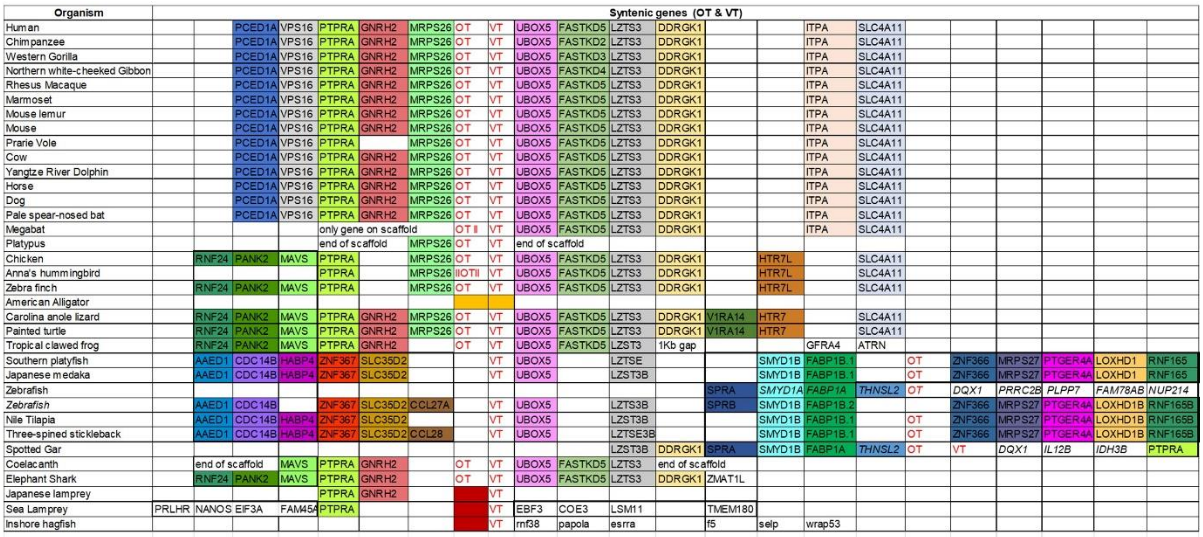

Organism	Syntenic genes (OT & VT)																					
Human			PCED1A	VPS16	PTPRA	GNRH2	MRPS26	OT	VT	UBOX5	FASTKD5	LZTS3	DDRGK1			ITPA	SLC4A11						
Chimpanzee			PCED1A	VPS16	PTPRA	GNRH2	MRPS26	OT	VT	UBOX5	FASTKD2	LZTS3	DDRGK1			ITPA	SLC4A11						
Western Gorilla			PCED1A	VPS16	PTPRA	GNRH2	MRPS26	OT	VT	UBOX5	FASTKD3	LZTS3	DDRGK1			ITPA	SLC4A11						
Northern white-cheeked Gibbon			PCED1A	VPS16	PTPRA	GNRH2	MRPS26	OT	VT	UBOX5	FASTKD4	LZTS3	DDRGK1			ITPA	SLC4A11						
Rhesus Macaque			PCED1A	VPS16	PTPRA	GNRH2	MRPS26	OT	VT	UBOX5	FASTKD5	LZTS3	DDRGK1			ITPA	SLC4A11						
Marmoset			PCED1A	VPS16	PTPRA	GNRH2	MRPS26	OT	VT	UBOX5	FASTKD5	LZTS3	DDRGK1			ITPA	SLC4A11						
Mouse lemur			PCED1A	VPS16	PTPRA	GNRH2	MRPS26	OT	VT	UBOX5	FASTKD5	LZTS3	DDRGK1			ITPA	SLC4A11						
Mouse			PCED1A	VPS16	PTPRA	GNRH2	MRPS26	OT	VT	UBOX5	FASTKD5	LZTS3	DDRGK1			ITPA	SLC4A11						
Prarie Vole			PCED1A	VPS16	PTPRA		MRPS26	OT	VT	UBOX5	FASTKD5	LZTS3	DDRGK1			ITPA	SLC4A11						
Cow			PCED1A	VPS16	PTPRA	GNRH2	MRPS26	OT	VT	UBOX5	FASTKD5	LZTS3	DDRGK1			ITPA	SLC4A11						
Yangtze River Dolphin			PCED1A	VPS16	PTPRA	GNRH2	MRPS26	OT	VT	UBOX5	FASTKD5	LZTS3	DDRGK1			ITPA	SLC4A11						
Horse			PCED1A	VPS16	PTPRA	GNRH2	MRPS26	OT	VT	UBOX5	FASTKD5	LZTS3	DDRGK1			ITPA	SLC4A11						
Dog			PCED1A	VPS16	PTPRA	GNRH2	MRPS26	OT	VT	UBOX5	FASTKD5	LZTS3	DDRGK1			ITPA	SLC4A11						
Pale spear-nosed bat			PCED1A	VPS16	PTPRA	GNRH2	MRPS26	OT	VT	UBOX5	FASTKD5	LZTS3	DDRGK1			ITPA	SLC4A11						
Megabat					only gene on scaffold			OT II	VT	UBOX5	FASTKD5	LZTS3	DDRGK1			ITPA	SLC4A11						
Platypus					end of scaffold		MRPS26	OT	VT	end of scaffold													
Chicken		RNF24	PANK2	MAVS	PTPRA		MRPS26	OT	VT	UBOX5	FASTKD5	LZTS3	DDRGK1		HTR7L		SLC4A11						
Anna's hummingbird					PTPRA		MRPS26	IIOTII	VT	UBOX5	FASTKD5	LZTS3	DDRGK1		HTR7L		SLC4A11						
Zebra finch		RNF24	PANK2	MAVS	PTPRA		MRPS26	OT	VT	UBOX5	FASTKD5	LZTS3	DDRGK1		HTR7L		SLC4A11						
American Alligator																							
Carolina anole lizard		RNF24	PANK2	MAVS	PTPRA	GNRH2	MRPS26	OT	VT	UBOX5	FASTKD5	LZTS3	DDRGK1	V1RA14	HTR7		SLC4A11						
Painted turtle		RNF24	PANK2	MAVS	PTPRA	GNRH2	MRPS26	OT	VT	UBOX5	FASTKD5	LZTS3	DDRGK1	V1RA14	HTR7		SLC4A11						
Tropical clawed frog		RNF24	PANK2	MAVS	PTPRA	GNRH2		OT	VT	UBOX5	FASTKD5	LZST3	1Kb gap			GFRA4	ATRN						
Southern platyfish		AAED1	CDC14B	HABP4	ZNF367	SLC35D2			VT	UBOX5		LZTSE			SMYD1B	FABP1B.1		OT	ZNF366	MRPS27	PTGER4A	LOXHD1	RNF165
Japanese medaka		AAED1	CDC14B	HABP4	ZNF367	SLC35D2			VT	UBOX5		LZST3B			SMYD1B	FABP1B.1		OT	ZNF366	MRPS27	PTGER4A	LOXHD1	RNF165
Zebrafish														SPRA	*SMYD1A*	*FABP1A*	*THNSL2*	*OT*	*DQX1*	*PRRC2B*	*PLPP7*	*FAM78AB*	*NUP214*
Zebrafish		AAED1	CDC14B		ZNF367	SLC35D2	CCL27A		VT	UBOX5		LZTS3B		SPRB	SMYD1B	FABP1B.2			ZNF366	MRPS27	PTGER4A	LOXHD1B	RNF165B
Nile Tilapia		AAED1	CDC14B	HABP4	ZNF367	SLC35D2			VT	UBOX5		LZST3B			SMYD1B	FABP1B.1		OT	ZNF366	MRPS27	PTGER4A	LOXHD1B	RNF165B
Three-spined stickleback		AAED1	CDC14B	HABP4	ZNF367	SLC35D2	CCL28		VT	UBOX5		LZTSE3B			SMYD1B	FABP1B.1		OT	ZNF366	MRPS27	PTGER4A	LOXHD1B	RNF165B
Spotted Gar												LZST3B	DDRGK1	SPRA	SMYD1B	FABP1A	*THNSL2*	OT	VT	*DQX1*	*IL12B*	*IDH3B*	PTPRA
Coelacanth		end of scaffold		MAVS	PTPRA	GNRH2		OT	VT	UBOX5	FASTKD5	LZTS3	end of scaffold										
Elephant Shark		RNF24	PANK2	MAVS	PTPRA	GNRH2		OT	VT	UBOX5	FASTKD5	LZTS3	DDRGK1	ZMAT1L									
Japanese lamprey					PTPRA	GNRH2			VT														
Sea Lamprey	PRLHR	NANOS	EIF3A	FAM45A	PTPRA				VT	EBF3	COE3	LSM11		TMEM180									
Inshore hagfish									VT	rnf38	papola	esrra		f5	selp	wrap53							

Supplementary Fig. 1: Microsynteny manual analysis for *OT* and *VT* genes. Colors denote orthologous genes. Detailed versions of the data with accession IDs, location, aliases, number of exons and a longer syntenic window, are in Supplementary Table 4a. Dark red shading, the gene never evolved in that lineage.

Organism	Syntenic Genes (OTR)													
Human	LHFPL4	SETD5		THUMPD3	SRGAP3	RAD18	OTR	CAV3	SSUH2	LMCD1	GRM7			EDEM1
Chimpanzee	LHFPL4	SETD5		THUMPD3	SRGAP3	RAD18	OTR	CAV3	SSUH2	LMCD1	GRM7			EDEM1
Western Gorilla	LHFPL4	SETD5		THUMPD3	SRGAP3	RAD18	OTR	CAV3	SSUH2	LMCD1	GRM7			EDEM1
Northern white-cheeked Gibbon	LHFPL4	SETD5		THUMPD3	SRGAP3	RAD18	OTR	CAV3	SSUH2	LMCD1	GRM7			EDEM1
Rhesus Macaque	LHFPL4	SETD5		THUMPD3	SRGAP3	RAD18	OTR	CAV3	SSUH2	LMCD1	GRM7			EDEM1
Marmoset	LHFPL4	SETD5		THUMPD3	SRGAP3	RAD18	OTR	CAV3	CCDC14	LMCD1	GRM7			EDEM1
Mouse lemur	LHFPL4	SETD5		THUMPD3	SRGAP3	RAD18	OTR	CAV3		LMCD1				EDEM1
Mouse	LHFPL4	SETD5		THUMPD3	SRGAP3	RAD18	OTR	CAV3	SSU2	LMCD1	GRM7			EDEM1
Prarie Vole	LHFPL4	SETD5		THUMPD3	SRGAP3	RAD18	OTR	CAV3	SSUH2	LMCD1	GRM7			EDEM1
Cow	LHFPL4	SETD5		THUMPD3	SRGAP3	RAD18	OTR	CAV3	SSUH2	LMCD1	GRM7			EDEM1
Yangtze River Dolphin	LHFPL4	SETD5		THUMPD3	SRGAP3	RAD18	OTR	CAV3	SSUH2	LMCD1	GRM7			
Horse	LHFPL4	SETD5		THUMPD3	SRGAP3	RAD18	OTR	CAV3	SSUH2	LMCD1	GRM7			EDEM1
Dog	LHFPL4	SETD5		THUMPD3	SRGAP3	RAD18	OTR	CAV3	SSUH2	LMCD1	GRM7			EDEM1
Pale spear-nosed bat	LHFPL4	SETD5		THUMPD3	SRGAP3	RAD18	OTR	CAV3	SSUH2	LMCD1	GRM7			EDEM1
Megabat	LHFPL4	SETD5		THUMPD3	SRGAP3	RAD18	OTR	CAV3	SSUH2	LMCD1	GRM7			EDEM1
**Platypus**	LHFPL4	SETD5		THUMPD3	SRGAP3	RAD18**	OTR		SSUH2	LMCD1				EDEM1
Chicken	IRAK2	VHL		THUMPD3	SRGAP3	RAD18	OTR	CAV3		LMCD1	GRM7			EDEM1
Anna's hummingbird	SEC13	VHL		THUMPD3	SRGAP3	RAD18	OTR	CAV3		LMCD1	GRM7			EDEM1
Zebra finch	IRAK2	VHL		THUMPD3	SRGAP3	RAD18	OTR	CAV3		LMCD1	GRM7			EDEM1
American Alligator	IRAK2	VHL		THUMPD3	SRGAP3	RAD18	OTR	CAV3		LMCD1	GRM7			EDEM1
Carolina anole lizard	IRAK2	VHL		THUMPD3	SRGAP3	RAD18	OTR	end of scaffold						EDEM1
Painted turtle	IRAK2	VHL		THUMPD3	SRGAP3	RAD18	OTR	CAV3	SSUH2	LMCD1	GRM7			EDEM1
Tropical clawed frog	IRAK2	VHL		THUMPD3	SRGAP3	RAD18	OTR	CAV3	SSUH2	LMCD1	GRM7			EDEM1
Southern platyfish	PBRM1		CSE1L	THUMPD3	SRGAP3	RAD18	OTR	CAV3	PARP3		GRM2A	TEX264A	FANCD2	
Japanese medaka	*CSE1L*	KCNB1		THUMPD3	SRGAP3	RAD18	OTRa	CAV3	PARP3		GRM2	TEX264	FANCD2	
Zebrafish		KCNB1	PTGIS	THUMPD3	SRGAP3	RAD18	OTRa	CAV3	PARP3		GRM2A	TEX264A	FANCD2	
Nile Tilapia	PBRM1		CSE1L	THUMPD3	SRGAP3	RAD18	OTRa	CAV3	PARP3		GRM2A	TEX264A	FANCD2	
Three-spined stickleback	CISH	HEMK1			SRGAP3	RAD18	OTRa	CAV3	PARP3		GRM2A	TEX264A	FANCD2	EDEM1
Spotted Gar	PBRM1	SMIM4	STAB1	NISCH		RAD18	OTR	CAV3	PARP3	RRP9	GRM2B	TEX264A	FANCD2	EDEM1
Coelacanth	TMEM208	VHL		THUMPD3	SRGAP3	RAD18	OTR	CAV3	SSUH2	LMCD1	GRM7			EDEM1
Elephant Shark	*SEMA3H*	*ZMYND1*	*RASSF1*	*TUSC2*		RAD18	OTR	CAV3	SSUH2	*GRIP2*	*SEC13*	*CAND2*	*CCDC174*	EDEM1
**Japanese lamprey**			GRIP2	THUMPD3	SRGAP3/2/1	TMEM5	OTR		SSUH2	LMCD1			SEMA3G/A/B/I	CACNA2D2/1
Sea Lamprey		VHL	GRIP1/2	THUMPD3	SRGAP2/3	TMEM5	OTR		SSUH2	LMO6	LMCD1	TFE3	SEMA3AA/AB/	*TEX264*

Supplementary Fig. 2: Microsynteny manual analysis for *OTR*. Colors denote orthologous genes. Detailed versions of the data with accession IDs, location, aliases, number of exons and a longer syntenic window, are in Supplementary Table 4b.

Organism	Syntenic genes (VTR1A)														
Human	GRIP1	~13 genes	TBK1	XPOT	C12orf56	C12orf66	SRGAP1	TMEM5	DPY19L2	VTR1A	PPM1H	MON2	USP15	FAM19A2	SLC16A7
Chimpanzee	GRIP1	~15 genes	TBK1	XPOT	C12Horf56	C12Horf66	SRGAP1	TMEM5	DPY19L2	VTR1A	PPM1H	MON2	USP15	FAM19A2	SLC16A7
Western Gorilla	GRIP1	~40 Mb	TBK1	XPOT	C12Horf56	C12Horf66	SRGAP1	TMEM5	DPY19L2	VTR1A	PPM1H	MON2	USP15	FAM19A2	SLC16A7
Northern white-cheeked Gibbon			TBK1	XPOT	C12Horf56	C12Horf66	SRGAP1	TMEM5	DPY19L2	VTR1A	PPM1H	MON2	USP15	FAM19A2	SLC16A7
Rhesus Macaque	GRIP1	~15 genes	TBK1	XPOT	C11Horf56	C11Horf66	SRGAP1	TMEM5	DPY19L2	VTR1A	PPM1H	MON2	USP15	FAM19A2	SLC16A7
Marmoset	GRIP1	~13 genes	TBK1	XPOT	C9H12orf56	C9H12orf66	SRGAP1	TMEM5	DPY19L2	VTR1A	PPM1H	MON2	USP15	FAM19A2	SLC16A7
Mouse lemur	GRIP1	~13 genes	TBK1	XPOT	C7H12orf56	C7H12orf66	SRGAP1	TMEM5	DPY19L2	VTR1A	PPM1H	MON2	USP15	FAM19A2	SLC16A7
Mouse			TBK1	XPOT			SRGAP1	TMEM5		VTR1A	PPM1H	MON2	USP15	FAM19A2	SLC16A7
Prarie Vole	GRIP1	~9 genes	TBK1	XPOT						VTR1A	PPM1H	MON2	USP15	FAM19A2	SLC16A7
Cow			TBK1	XPOT	C5H12orf56	C5H12orf66	SRGAP1	TMEM5	DPY19L2	VTR1A	PPM1H	MON2	USP15	FAM19A2	SLC16A7
Yangtze River Dolphin					end of scaffold		SRGAP1	TMEM5	DPY19L2	VTR1A	PPM1H	MON2	end of scaffold		
Horse	GRIP1	~13 genes	TBK1	XPOT	C6H12orf56	C6H12orf66	SRGAP1	TMEM5	DPY19L2	VTR1A	PPM1H	MON2	USP15	FAM19A2	SLC16A7
Dog	GRIP1	~13 genes	TBK1	XPOT		CUNH12orf66	SRGAP1	TMEM5	DPY19L2	VTR1A	PPM1H	MON2	USP15	FAM19A2	SLC16A7
Pale spear-nosed bat	GRIP1	~13 genes	TBK1	XPOT	C2H12orf56	C2H12orf66	SRGAP1	RXYLT1		VTR1A	PPM1H	MON2	USP15	TAFA2	SLC16A7
Megabat	GRIP1	~13 genes	TBK1	XPOT	C6H12orf56	CUNH12orf66	SRGAP1	RXYLT1		VTR1A	end of scaffold				
**Platypus**								only gene on contig		VTR1A	only gene on contig				
Chicken	GRIP1	~10 genes	TBK1	XPOT		C1H12orf66	SRGAP1	TMEM5		VTR1A	PPM1H	MON2	USP15	FAM19A2	SLC16A7
Anna's hummingbird	GRIP1	~10 genes	TBK1	XPOT	RPL18A	C5H12orf66	SRGAP1	TMEM5		VTR1A	PPM1H	MON2	USP15	FAM19A2	SLC16A7
Zebra finch	GRIP1	~10 genes	TBK1	XPOT		C1AH12orf66	SRGAP1	TMEM5		VTR1A	PPM1H	MON2	USP15	FAM19A2	SLC16A7
American Alligator	GRIP1	~15 genes	TBK1	XPOT		CUNH12orf66	SRGAP1	TMEM5		VTR1A	PPM1H	MON2	USP15	FAM19A2	SLC16A7
Carolina anole lizard			TBK1	XPOT	RPL18A	C5H12orf66	SRGAP1	TMEM5		VTR1A	PPM1H	MON2	USP15	FAM19A2	SLC16A7
Painted turtle			TBK1	XPOT			SRGAP1	TMEM5		VTR1A	PPM1H	MON2	USP15	FAM19A2	TWIST1
Tropical clawed frog				CDK17	C12orf63	NEDD1	TMPO	SLC25A3	NUAK1	VTR1Aa	PPM1H	MON2	RPS16	OTOGL	PTPRQ
Southern platyfish					CAT	IFITM5	PTDSS2	TMEM168	BMT2	VTR1Aa	PPM1H	MON2	FBLN1	WNT7BB	PPARAB
Japanese medaka				PTDSS2	TMEM168B	BMT2		FOXP2L	CCDC42	VTR1Aa	PPM1H	MON2	SLC6A13	AKR1D1	FBLN1
Zebrafish				PPP1R3A	BMT2	TMEM168B	PTDSS2	CDKN1C	IFITM5	VTR1Aa	PPM1H	MON2	SLC6A13	AKR1D1	PPARAB
Nile Tilapia					IFITM5	CDKN1CB	PTDSS2	TMEM168	BMT2	VTR1Aa	PPM1H	MON2	AKR1D1	WNT7BB	PPARAB
Three-spined stickleback					FAM96A	CALML4B	CLN6B	FEM1B	ITGA11B	VTR1Aa	PPM1H	MON2	FBLN1	WNT7BB	PPARAB
Spotted Gar	GRIP1	~20 Mb		FAM180A	MTPN	CLG8H12orf6	SRGAP1			VTR1A	PPM1H	MON2	USP15	KDM5A	RAD52
Coelacanth								end of scaffold		VTR1A	PPM1H	MON2	USP15	FAM19A2	
Elephant Shark			TBK1	XPOT	C12orf56	C12orf66	SRGAP1	TMEM5		VTR1A	PPM1H	MON2	USP15	FAM19A2	
**Japanese lamprey**	GRIP1/2		LTA4H	TCAF2	CDK17/16/1	DNAH3/7/12/1	SRGAP1/2/3			VTR1A	KIAA1033	ALDH1L2			
Sea Lamprey			ABT1	TTC25	CAMTA2	DNAH3/1/7	SRGAP1			VTR1A	KIAA1034	ALDH1L2			

Supplementary Fig. 3: Microsynteny manual analysis for *VTR1A*. Colors denote orthologous genes. Detailed versions of the data with accession IDs, location, aliases, number of exons and a longer syntenic window, are in Supplementary Table 4c.

Organism	Syntenic Genes (VTR1B)										
Human	PM20D1	SLC26A9	RAB7B	CTSE	C1orf186	VTR1B	FAM72A	SRGAP2	IKBKE	RASSF5	EIF2D
Chimpanzee	PM20D1	SLC26A9	RAB7B	CTSE	C1Horf186	VTR1B	FAM72A	SRGAP2	IKBKE	RASSF5	EIF2D
Western Gorilla	*IKBKE*	*SRGAP2*	RAB7B	CTSE	C1Horf186	VTR1B	FAM72A	*SLC26A9*	*PM20D1*	*SLC41A1*	*RAB29*
Northern white-cheeked Gibbon	PM20D1	SLC26A9		CTSE	C5H1orf186	VTR1B	FAM72A	SRGAP2	IKBKE	RASSF5	EIF2D
Rhesus Macaque	PM20D1	SLC26A9		CTSE	C5H1orf186	VTR1B	FAM72A	SRGAP2	IKBKE	RASSF5	EIF2D
Marmoset	PM20D1	SLC26A9		CTSE	C19H1orf186	VTR1B	FAM72A	SRGAP2	IKBKE	RASSF5	EIF2D
Mouse lemur	PM20D1	SLC26A9	RAB7B	CTSE	C27H1orf186	VTR1B	FAM72A	SRGAP2	IKBKE	RASSF5	EIF2D
Mouse	PM20D1	SLC26A9	RAB7B	CTSE		VTR1B	FAM72A	SRGAP2	IKBKE	RASSF5	EIF2D
Prarie Vole	PM20D1	SLC26A9		CTSE		VTR1B	FAM72A	SRGAP2	IKBKE	RASSF5	EIF2D
Cow	PM20D1	SLC26A9	RAB7B	CTSE	C16H1orf186	VTR1B	FAM72A	SRGAP2	IKBKE	RASSF5	EIF2D
Yangtze River Dolphin	PM20D1	SLC26A9	RAB7B		LOC	VTR1B	FAM72A	SRGAP2	IKBKE	RASSF5	EIF2D
Horse	PM20D1	SLC26A9	RAB7B	CTSE	C5H1orf186	VTR1B		SRGAP2	IKBKE	RASSF5	EIF2D
Dog	PM20D1	SLC26A9	RAB7B	CTSE	C38H1orf186	VTR1B	FAM72A	SRGAP2	IKBKE	RASSF5	EIF2D
Pale spear-nosed bat	PM20D1	SLC26A9	RAB7B	CTSE	RHEX	VTR1B	FAM72A	SRGAP2	IKBKE	RASSF5	EIF2D
Megabat	PM20D1	SLC26A9	RAB7B	CTSE	LOC	VTR1B	FAM72A	SRGAP2	IKBKE	RASSF5	EIF2D
Platypus	TMCC2	SLC26A9	RAB7B			VTR1B		SRGAP2	IKBKE	IL10	YOD1
Chicken	PM20D1		RAB7B	CTSE		VTR1B	FAM72A	SRGAP2	IKBKE	RASSF5	EIF2D
Anna's hummingbird	PM20D1	SLC26A9	RAB7B	CTSE		VTR1B	FAM72A	SRGAP2	IKBKE	RASSF5	EIF2D
Zebra finch	PM20D1	SLC26A9	RAB7B	CTSE		VTR1B	SLC45A3	DDX20	KCND3	CTTNBP2N2L	WNT2B
American Alligator	PM20D1	SLC26A9	RAB7B	CTSE	CUNH1orf186	VTR1B	FAM72A	SRGAP2	IKBKE	RASSF5	EIF2D
Carolina anole lizard	PM20D1	SLC26A9	RAB7B	CTSE		VTR1B	FAM72A	SRGAP2	IKBKE	RASSF5	EIF2D
Painted turtle	PM20D1	SLC26A9	RAB7B	CTSE		VTR1B	FAM72A	SRGAP2	IKBKE	RASSF5	EIF2D
Tropical clawed frog	PM20D1	SLC26A9	RAB7B	CTSE		VTR1B	FAM72A	SRGAP2	IKBKE	RASSF5	EIF2D
Southern platyfish	MYOG	*PPFIA4*	*TFEB*	*TMEM18*	FOXP4		FAM72B	SRGAP2	IKBKE	RASSF5	
Japanese medaka	PPFIA4	TMEM183A	TFEB	MDFIC	FOXP4		FAM72A	SRGAP2	IKBKE	RASSF5	
Zebrafish	PPFIA4	TMEM183B	TFEB	MDFIC	FOXP4		FAM72A	SRGAP2	IKBKE	RASSF5	
Nile Tilapia	PPFIA4	TMEM183A	TFEB	MDFIC	FOXP4		FAM72B	SRGAP2	IKBKE	RASSF5	
Three-spined stickleback	PPFIA4	TMEM183A	TFEB	MDFIC	FOXP4		FAM72A	SRGAP2	IKBKE	RASSF5	
Spotted Gar	PRELP	CMRF35L9	TFEB	MDFI	FOXP4		FAM72A	SRGAP2	IKBKE	MUC2	RASSF5
Coelacanth	PM20D1	SLC26A9	RAB7B	CTSE		VTR1B	FAM72A	SRGAP2	IKBKE	RASSF5	EIF2D
Elephant Shark	PROK1	KCNC4	FOXP4	MDFI	TFEB	VTR1B	FAM72A	SRGAP2	IKBKE	SYPL2	ATXN7L2
Japanese lamprey											
Sea Lamprey											

Supplementary Fig. 4: Microsynteny manual analysis for *VTR1B*. Colors denote orthologous genes. Detailed versions of the data with accession IDs, location, aliases, number of exons and a longer syntenic window, are in Supplementary Table 4d. Dark red shading, the gene never evolved in that lineage; light red shading, loss of the VTR1B gene.

Organism	Syntenic genes (VTR2A)										
Human	GXYLT1	PDZRN4	CNTN1 II	NRCAML	PNPLA8		THAP5	DNAJB9	IMMP2L	LRRN3	DOCK4
Chimpanzee	GXYLT1	PDZRN4	CNTN1 II	NRCAML	PNPLA8		THAP5	DNAJB9	IMMP2L	LRRN3	DOCK4
Western Gorilla	GXYLT1	PDZRN4	CNTN1 II	NRCAML	PNPLA8		THAP5	DNAJB9	IMMP2L	LRRN3	DOCK4
Northern white-cheeked Gibbon	GXYLT1	PDZRN4	CNTN1 II	NRCAML	PNPLA8		THAP5	DNAJB9	IMMP2L	LRRN3	DOCK4
Rhesus Macaque	GXYLT1	PDZRN4	CNTN1 II	NRCAML	PNPLA8		THAP5	DNAJB9	IMMP2L	LRRN3	DOCK4
Marmoset	GXYLT1	PDZRN4	CNTN1 II	NRCAML	PNPLA8		THAP5	DNAJB9	IMMP2L	LRRN3	DOCK4
Mouse lemur	GXYLT1	PDZRN4	CNTN1 II	NRCAML	PNPLA8		THAP5	DNAJB9	IMMP2L	LRRN3	DOCK4
Mouse	GXYLT1	PDZRN4	CNTN1 II	NRCAML	PNPLA8		THAP5	DNAJB9	IMMP2L	LRRN3	DOCK4
Prarie Vole	GXYLT1	PDZRN4	CNTN1 II	NRCAML	PNPLA8			DNAJB9	IMMP2L	LRRN3	DOCK4
Cow	GXYLT1	PDZRN4	CNTN1 II	NRCAML	PNPLA8				IMMP2L II	LRRN3	DOCK4
Yangtze River Dolphin	GXYLT1 II			NRCAML	PNPLA8						DOCK4
Horse	GXYLT1	PDZRN4	CNTN1 II	NRCAML	PNPLA8				IMMP2L II	LRRN3	DOCK4
Dog	GXYLT1	PDZRN4	CNTN1 II	NRCAML	PNPLA8				IMMP2L II	LRRN3	DOCK4
Pale spear-nosed bat	GXYLT1	PDZRN4	CNTN1 II	NRCAM	PNPLA8 II				IMMP2L	LRRN3	DOCK4
Megabat	GXYLT1	PDZRN4	CNTN1 II						IMMP2L	LRRN3	DOCK4
**Platypus**	GXYLT1	PDZRN4 II		*PNPLA8*	*NRCAM*		*IFRD1*		II IMMP2L		DOCK4
Chicken	GXYLT1	PDZRN4	CNTN1	NRCAM	PNPLA8	VTR2A	THAP5	DNAJB9	IMMP2L	LRRN3	DOCK4
Anna's hummingbird	**end of scaffold**		CNTN1	NRCAM	PNPLA8	VTR2A	**end of scaffold**				
Zebra finch	GXYLT1	PDZRN4	CNTN1	NRCAM	PNPLA8	VTR2A	THAP5	DNAJB9	*LRRN3*	*IMMP2L*	DOCK4
American Alligator	GXYLT1	PDZRN4	CNTN1	NRCAM	PNPLA8	VTR2A	THAP5	DNAJB9	IMMP2L	LRRN3	DOCK4
Carolina anole lizard	GXYLT1	PDZRN4	CNTN1	NRCAM	PNPLA8	VTR2A			IMMP2L	LRRN3	DOCK4
Painted turtle	GXYLT1	PDZRN4	CNTN1	NRCAM	PNPLA8	VTR2A	THAP5	DNAJB9	IMMPL2	LRRN3	DOCK4
Tropical clawed frog	GXYLT1	PDZRN4	CNTN1	NRCAM	PNPLA8	VTR2A	THAP5	DNAJB9	IMMPL2	LRRN3	DOCK4
Southern platyfish	GXYLT1	PDZRN4	CNTN1B	NRCAMA	PNPLA8	VTR2Aa	LOC	DNAJB9B	AKR1B1	TFEC	
Japanese medaka	GXYLT1B	PDZRN4	CNTN1B	NRCAMA	PNPLA8	VTR2Aa	THAP5	DNAJB9B	AKR1B1	TFEC	MDFIC
Zebrafish	LDHBA	KISS2	CNTN1B	NRCAMA	PNPLA8		THAP5	DNAJB9B	AKR1B1	TFEC	MDFIC
Nile Tilapia	GXYLT1B	PDZRN4	CNTN1B	NRCAMA	PNPLA8		THAP5	DNAJB9B	AKR1B1	TFEC	MDFIC
Three-spined stickleback	GXYLT1B	PDZRN4	CNTN1B	NRCAMA	PNPLA8		CCDC8	IRF5	TNPO3	OPN1SW1	CALUA
Spotted Gar	GXYLT1	PDZRN4	CNTN1	NRCAM	PNPLA8	VTR2A	THAP5	DNAJB9			
Coelacanth				NRCAM	PNPLA8		THAP5	DNAJB9	**end of scaffold**		
Elephant Shark	NAMPT	EFCAB6	SLC6A1		PNPLA8	VTR2A	THAP5	DNAJB9	*LRRN3*	*IMMPL2*	*DOCK4*
**Japanese lamprey**			CNTN2/5	NRCAML		VTR2A			IMMP2L	LRRN1/3/2	
Sea Lamprey			CNTN2	NRCAML		VTR2A			IMMP2L	LRRN1/3/2	

Supplementary Fig. 5: Microsynteny manual analysis for *VTR2A*. Colors denote orthologous genes. Detailed versions of the data with accession IDs, location, aliases, number of exons and a longer syntenic window, are in Supplementary Table 4c. Light red shading, loss of the VTR2A gene.

Organism	Syntenic Genes (VTR2B)															
Human	BHLHE40	ITPR1	SUMF1	SETMAR	LRRN1			CRBN	TRNT1	IL5RA	CNTN4	CNTN6	CHL1			
Chimpanzee	BHLHE40	ITPR1	SUMF1	SETMAR	LRRN1			CRBN	TRNT1	IL5RA	CNTN4	CNTN6	CHL1			
Western Gorilla	BHLHE40	ITPR1	SUMF1	SETMAR	LRRN1			CRBN	TRNT1	IL5RA	CNTN4	CNTN6	CHL1			
Northern white-cheeked Gibbon	BHLHE40	ITPR1	SUMF1	SETMAR	LRRN1			CRBN	TRNT1	IL5RA	CNTN4	CNTN6	CHL1			
Rhesus Macaque	BHLHE40	ITPR1	SUMF1	SETMAR	LRRN1			CRBN	TRNT1	IL5RA	CNTN4	CNTN6	CHL1			
Marmoset	BHLHE40	ITPR1	SUMF1	SETMAR	LRRN1			CRBN	TRNT1	IL5RA	CNTN4	CNTN6	CHL1			
Mouse lemur	BHLHE40	ITPR1	SUMF1	MARK3	LRRN1			CRBN	TRNT1	IL5RA	CNTN4	GRM7	DRD1			
Mouse	BHLHE40	ITPR1	SUMF1	SETMAR	LRRN1			CRBN	TRNT1	IL5RA	CNTN4	CNTN6	CHL1			
Prarie Vole	BHLHE40	ITPR1	SUMF1		LRRN1			CRBN	TRNT1	IL5RA	CNTN4	CNTN6	CHL1			
Cow	BHLHE40	ITPR1	SUMF1	SETMAR	LRRN1			CRBN	TRNT1	IL5RA	CNTN4	CNTN6	CHL1			
Yangtze River Dolphin		end of scaffold		SETMAR	LRRN1			CRBN	TRNT1	IL5RA	CNTN4	CNTN6	CHL1			
Horse	BHLHE40	ITPR1	SUMF1	SETMAR	LRRN1			CRBN	TRNT1	IL5RA	CNTN4	CNTN6	CHL1			
Dog	BHLHE40	ITPR1	SUMF1	SETMAR	LRRN1			CRBN	TRNT1	IL5RA	CNTN4	CNTN6	CHL1			
Pale spear-nosed bat	BHLHE40	ITPR1	SUMF1	LOC	LRRN1	~7 genes		CRBN	TRNT1	IL5RA	CNTN4	CNTN6	CHL1			
Megabat	BHLHE40	ITPR1	SUMF1	SETMAR	LRRN1			CRBN	TRNT1	IL5RA	CNTN4	end scaffold				
**Platypus**	BHLHE40	ITPR1	LOC	LOC	LRRN1			CRBN	TRNT1	SLC38A8	OSGIN1	SMPD3	PRMT7			
Chicken	BHLHE40	ITPR1	SUMF1	SETMAR	LRRN1			CRBN	TRNT1	IL5RA	CNTN4	CNTN6	CHL1			
Anna's hummingbird	BHLHE40	ITPR1	SUMF1		LRRN1			CRBN	TRNT1	IL5RA	CNTN4	CNTN6				
Zebra finch	BHLHE40	ITPR1	SUMF1		LRRN1			CRBN	TRNT1	IL5RA	CNTN4	CNTN6	CHL1			
American Alligator	BHLHE40	ITPR1	SUMF1		LRRN1			CRBN	TRNT1	IL5RA	CNTN4	CNTN6	CHL1			
Carolina anole lizard	BHLHE40	ITPR1	SUMF1		LRRN1			CRBN	TRNT1	IL5RA	CNTN4	CNTN6	CHL1			
Painted turtle	BHLHE40	ITPR1	SUMF1		LRRN1			CRBN	TRNT1	IL5RA	CNTN4	CNTN6				
Tropical clawed frog	BHLHE40	ITPR1	SUMF1	SETMAR	LRRN1			CRBN	TRNT1	IL5RA	CNTN4					
Southern platyfish	BHLHE40	ITPR1B		SETMAR	LRRN1				TRNT1				FRMD4BA	MITFA	EEVS	FOXP1B
Japanese medaka	BHLHE40	ITPR1B		SETMAR	LRRN1		VTR2Ba		TRNT1	*PRA1*	ARL6IP5A		FRMD4BA	MITFA	CAMKV	MST1R
Zebrafish	*DECC1B*	ITPR1A	SUMF1		LRRN1											
Nile Tilapia	BHLHE40	ITPR1B		SETMAR	LRRN1		VTR2Ba		TRNT1		ARL6IP5A		FRMD4BA	MITFA	CCDC15	DDX4
Three-spined stickleback	*TNFRSF1*	*GLYCTK*			LRRN1		VTR2Ba		TRNT1		ARL6IP5A		FRMD4BA	MITFA	EEVS	FOXP1B
Spotted Gar	BHLHE40	ITPR1	SUMF1	SETMAR	LRRN1	*CRBN*	VTR2B		TRNT1	VHL	TATDN2	CCDC174	FGD5A			
Coelacanth		TMEM1	INF2 ||			LOC	VTR2B	CRBN	TRNT1		CNTN4	end scaffold				
Elephant Shark		ITPR1	SUMF1		LRRN1	SNX6	VTR2B	CRBN	TRNT1	CHL1	CNTN4	end scaffold				
**Japanese lamprey**	SLC16A7	RHOA	EMC3		SORT1		VTR2B	CRBN	SLC25A	DMTF1	MANF		FRMD4B/A			
Sea Lamprey	SLC16A1	RHOA	EMC3	GPX2	SORCS1		VTR2B	CRBN		DMTF1	MANF	FAM107B	FRMD4B/A			

Supplementary Fig. 6: Microsynteny manual analysis for *VTR2B*. Colors denote orthologous genes. Detailed versions of the data with accession IDs, location, aliases, number of exons and a longer syntenic window, are in Supplementary Table 4b. Light red shading, loss of the VTR2B gene.

Organism	Syntenic genes (VTR2C)															
Human	SRPK3	IDH3G	SSR4	PDZD4	L1CAM	LCA10	VTR2C					ARHGAP4	NAA10	RENBP	HCFC1	TMEM187
Chimpanzee	SRPK3	IDH3G	SSR4	PDZD4	L1CAM	LCA10	VTR2C					ARHGAP4	NAA10	RENBP	HCFC1	MECP2
Western Gorilla	SRPK3	IDH3G	SSR4	PDZD4			VTR2C					ARHGAP4	NAA10	RENBP	HCFC1	TMEM187
Northern white-cheeked Gibbon		IDH3G	SSR4	PDZD4	L1CAM		VTR2C					ARHGAP4	NAA10	RENBP	HCFC1	TMEM187
Rhesus Macaque		IDH3G	SSR4	PDZD4	L1CAM		VTR2C					ARHGAP4	NAA10	RENBP	HCFC1	TMEM187
Marmoset	SRPK3	IDH3G	SSR4	PDZD4	L1CAM		VTR2C					ARHGAP4	NAA10	RENBP	HCFC1	TMEM187
Mouse lemur	SRPK3	IDH3G	SSR4	PDZD4	L1CAM	LCA10	VTR2C					ARHGAP4	NAA10	RENBP	HCFC1	TMEM187
Mouse	SRPK3	IDH3G	SSR4	PDZD4	L1CAM		VTR2C					ARHGAP4	NAA10	RENBP	HCFC1	
Prarie Vole	SRPK3	IDH3G	SSR4	PDZD4	L1CAM		VTR2C					ARHGAP4	NAA10	RENBP	HCFC1	end of scaffold
Cow	SRPK3	IDH3G	SSR4	PDZD4	L1CAM		VTR2C					ARHGAP4	NAA10	RENBP	HCFC1	TMEM187
Yangtze River Dolphin	SRPK3	IDH3G	SSR4	PDZD4	L1CAM		VTR2C					ARHGAP4	NAA10	RENBP	HCFC1	TMEM187
Horse	SRPK3	IDH3G	SSR4	PDZD4	L1CAM		VTR2C					ARHGAP4	NAA10	RENBP	HCFC1	TMEM187
Dog	SRPK3	IDH3G	SSR4	PDZD4	L1CAM		VTR2C					ARHGAP4	NAA10	RENBP	HCFC1	TMEM187
Pale spear-nosed bat	SRPK3	IDH3G	SSR4	PDZD4	L1CAM		VTR2C					ARHGAP4	NAA10	RENBP	HCFC1	TMEM187
Megabat	SRPK3	IDH3G	SSR4	PDZD4	L1CAM		VTR2C					ARHGAP4	NAA10	RENBP	HCFC1	TMEM187
**Platypus**					end of scaffold		VTR2C	CAV2				ARHGAP4	end of scaffold			
Chicken																
Anna's hummingbird																
Zebra finch																
American Alligator	SRPK3	IDH3G	SSR4	*L1CAM*	*PDZD4*		?			*NUDT16*	*NAA10*	*ARHGAP4*	TFE3	CCDC12	ATP2B3	
Carolina anole lizard		end of scaffold		PDZD4			VTR2C	end of scaffold								
Painted turtle	HAUS7	FLNA	SSR4	PDZD4	L1CAM		VTR2C	CAV2		*ARHGAP4*	*NUDT16*	*NAA10*	RENBP	HCFC1	RAB7A	TMEM187
Tropical clawed frog	HCFC1	SLC31A2	TMEM187	IRAK1	MECP2	OPN1LW	VTR2C	BRINP1	ASTN2	TRIM32	PAPPA	CAV2	ARHGAP	NAA10	NUDT16	RENBP
Southern platyfish	FAM3A	IDH3G					VTR2Ca	*SSR4*	LOC			ARHGAP4A	NAA10			GALNT6
Japanese medaka	FAM3A	IDH3G					VTR2Ca	*SSR4*	LOC			ARHGAP4A	NAA10	CERKL	G6PD	GALNT6
Zebrafish	FAM3A	IDH3G					VTR2Ca	*SSR4*	SRGAP3				NAA10	CERK	G6PD	GALNT6
Nile Tilapia	FAM3A	IDH3G					VTR2Ca	*SSR4*	LOC			ARHGAP4A	NAA10			GALNT6
Three-spined stickleback	FAM3A	IDH3G					VTR2Ca					ARHGAP4A		CASP9	NOL9	ZBTB48
Spotted Gar					end of scaffold		VTR2C	end of scaffold								
Coelacanth		BCAP31	SLC6A8	PDZD4	L1CAM		VTR2C	end of scaffold								
Elephant Shark																
**Japanese lamprey**																
Sea Lamprey																

Supplementary Fig. 7: Microsynteny manual analysis for *VTR2C*. Colors denote orthologous genes. Detailed versions of the data with accession IDs, location, aliases, number of exons and a longer syntenic window, are in Supplementary Tables 4e. Dark red shading, the gene never evolved in that lineage; light red shading, loss of a gene.

Supplementary Information-References

46. Bakos, J., Srancikova, A., Havranek, T. & Bacova, Z. Molecular Mechanisms of Oxytocin Signaling at the Synaptic Connection. *Neural plasticity* vol. 2018 4864107 (2018).

47. Gimpl, G. & Fahrenholz, F. The oxytocin receptor system: Structure, function, and regulation. *Physiological Reviews* vol. 81 629–683 (2001).

48. Brownstein, M. J., Russell, J. T. & Gainer, H. Synthesis, transport, and release of posterior pituitary hormones. *Science* vol. 207 373–378 (1980).

49. Renaud, L. P. & Bourquet, C. W. Neurophysiology and neuropharmacology of hypothalamic magnocellular neurons secreting vasopressin and oxytocin. *Progress in Neurobiology* vol. 36 131–169 (1991).

50. Melmed, S. *The Pituitary*. *The Pituitary* (Elsevier Inc., 2011). doi:10.1016/C2009-0-61488-4.

51. Korlach, J. *et al.* De novo PacBio long-read and phased avian genome assemblies correct and add to reference genes generated with intermediate and short reads. *Gigascience* **6**, (2017).

52. Hezroni, H. *et al.* Principles of Long Noncoding RNA Evolution Derived from Direct Comparison of Transcriptomes in 17 Species. *Cell Rep.* **11**, 1110–1122 (2015).

53. Stimpson, K. M. *et al.* Telomere Disruption Results in Non-Random Formation of De Novo Dicentric Chromosomes Involving Acrocentric Human Chromosomes. **6**, (2010).

54. Barra, V. & Fachinetti, D. The dark side of centromeres: types, causes and consequences of structural abnormalities implicating centromeric DNA. *Nat. Commun.* (2018) doi:10.1038/s41467-018-06545-y.

55. Merkin, J., Russell, C., Chen, P. & Burge, C. B. Evolutionary dynamics of gene and isoform regulation in mammalian tissues. *Science (80-.).* **338**, 1593–1599 (2012).

56. Barbosa-Morais, N. L. *et al.* The Evolutionary Landscape of Alternative Splicing in Vertebrate Species. *Science (80-.).* **338**, 1587–1593 (2012).

57. Holmes, C. L., Landry, D. W. & Granton, J. T. Science review: Vasopressin and the cardiovascular system part 1 - Receptor physiology. *Critical Care* vol. 7 427–434 (2003).

58. Tan, F. *et al.* Molecular Cloning and Functional Characterization of a Vasotocin Receptor Subtype That Is Expressed in the Shell Gland and Brain of the Domestic Chicken1. *Biol. Reprod.* **62**, 8–15 (2000).

59. Strakova, Z. & Soloff, M. S. Coupling of oxytocin receptor to G proteins in rat myometrium during labor: Gi receptor interaction. *Am. J. Physiol.* **272**, E870-6 (1997).

60. Osváth, S. & Gruebele, M. Proline can have opposite effects on fast and slow protein folding phases. *Biophys. J.* **85**, 1215–1222 (2003).

61. Verty, A. N. A., McFarlane, J. R., McGregor, I. S. & Mallet, P. E. Evidence for an interaction between CB1 cannabinoid and oxytocin receptors in food and water intake. *Neuropharmacology* **47**, 593–603 (2004).

62. Arthur, P., Taggart, M. J., Zielnik, B., Wong, S. & Mitchell, B. F. Relationship between gene expression and function of uterotonic systems in the rat during gestation, uterine activation and both term and preterm labour. *J. Physiol.* **586**, 6063–6076 (2008).

63. Marroni, S. S. *et al.* Neuroanatomical and cellular substrates of hypergrooming induced by microinjection of oxytocin in central nucleus of amygdala, an experimental model of compulsive behavior. *Mol. Psychiatry* **12**, 1103–1117 (2007).

64. Jankowski, M. *et al.* Oxytocin in cardiac ontogeny. *Proc. Natl. Acad. Sci. U. S. A.* **101**, 13074–13079 (2004).

65. Leng, G., Meddle, S. L. & Douglas, A. J. Oxytocin and the maternal brain. *Current Opinion in Pharmacology* vol. 8 731–734 (2008).

66. Witt, D. M. & Insel, T. R. Increased Fos Expression in Oxytocin Neurons Following Masculine Sexual Behavior. *J. Neuroendocrinol.* **6**, 13–18 (1994).

67. Insel, T. R. & Hulihan, T. J. A Gender-Specific Mechanism for Pair Bonding: Oxytocin and Partner Preference Formation in Monogamous Voles. *Behav. Neurosci.* **109**, 782–789 (1995).

68. Bosch, O. J., Meddle, S. L., Beiderbeck, D. I., Douglas, A. J. & Neumann, I. D. Brain oxytocin correlates with maternal aggression: Link to anxiety. *J. Neurosci.* **25**, 6807–6815 (2005).

69. Larrazolo-López, A. *et al.* Vaginocervical stimulation enhances social recognition memory in rats via oxytocin release in the olfactory bulb. *Neuroscience* **152**, 585–593 (2008).

70. Petersson, M., Alster, P., Lundeberg, T. & Uvnäs-Moberg, K. Oxytocin causes a long-term decrease of blood pressure in female and male rats. *Physiol. Behav.* **60**, 1311–1315 (1996).

71. Elabd, S. K., Sabry, I., Hassan, W. B., Nour, H. & Zaky, K. Possible neuroendocrine role for oxytocin in bone remodeling. *Endocr. Regul.* **41**, 131–41 (2007).

72. Magalhaes, J. K. R. S. *et al.* Oxytocin pretreatment decreases oxytocin-induced myometrial contractions in pregnant rats in a concentration-dependent but not time-dependent manner. *Reprod. Sci.* **16**, 501–508 (2009).

73. Wu, C. L., Hung, C. R., Chang, F. Y., Pau, K. Y. F. & Wang, P. S. Pharmacological effects of oxytocin on gastric emptying and intestinal transit of a non-nutritive liquid meal in female rats. *Naunyn. Schmiedebergs. Arch. Pharmacol.* **367**, 406–413 (2003).

74. Yang, J. *et al.* Effect of oxytocin on acupuncture analgesia in the rat. *Neuropeptides* **41**, 285–292 (2007).

75. Jirikowski, G. F., Caldwell, J. D., Pedersen, C. A. & Stumpf, W. E. Estradiol influences oxytocin-immunoreactive brain systems. *Neuroscience* **25**, 237–248 (1988).

76. Lukas, M. *et al.* The neuropeptide oxytocin facilitates pro-social behavior and prevents

social avoidance in rats and mice. *Neuropsychopharmacology* **36**, 2159–2168 (2011).

77. Filippi, S. *et al.* Role of oxytocin in the ejaculatory process. *J. Endocrinol. Invest.* **26**, 82–6 (2003).

78. Marlin, B. J., Mitre, M., D'Amour, J. A., Chao, M. V. & Froemke, R. C. Oxytocin enables maternal behaviour by balancing cortical inhibition. *Nature* **520**, 499–504 (2015).

79. Klatt, J. D. & Goodson, J. L. Oxytocin-like receptors mediate pair bonding in a socially monogamous songbird. *Proc. R. Soc. B Biol. Sci.* **280**, (2013).

80. Goodson, J. L., Lindberg, L. & Johnson, P. Effects of central vasotocin and mesotocin manipulations on social behavior in male and female zebra finches. *Horm. Behav.* **45**, 136–143 (2004).

81. Jonaidi, H., Oloumi, M. M. & Denbow, D. M. Behavioral effects of intracerebroventricular injection of oxytocin in birds. *Physiol. Behav.* **79**, 725–729 (2003).

82. Goodson, J. L., Schrock, S. E. & Kingsbury, M. A. Oxytocin mechanisms of stress response and aggression in a territorial finch. *Physiol. Behav.* **141**, 154–163 (2015).

83. Carr, J. L., Messinger, M. A. & Patton, G. M. Nesting Behavior in Three-Toed Box Turtles (Terrapene carolina triunguis) Following Oxytocin-Induced Oviposition . *Chelonian Conserv. Biol.* **7**, 124–128 (2008).

84. Jean-Luc, D. R. *et al.* Vasotocin and mesotocin stimulate the biosynthesis of neurosteroids in the frog brain. *J. Neurosci.* **26**, 6749–6760 (2006).

85. Goodson, J. L., Evans, A. K. & Bass, A. H. Putative isotocin distributions in sonic fish: Relation to vasotocin and vocal-acoustic circuitry. *J. Comp. Neurol.* **462**, 1–14 (2003).

86. Goodson, J. L. & Bass, A. H. Forebrain peptides modulate sexually polymorphic vocal circuitry. *Nature* **403**, 769–772 (2000).

87. Zimmermann, F. F., Gaspary, K. V., Siebel, A. M. & Bonan, C. D. Oxytocin reversed MK-801-induced social interaction and aggression deficits in zebrafish. *Behav. Brain Res.* **311**, 368–374 (2016).

88. Wee, C. L. *et al.* Zebrafish oxytocin neurons drive nocifensive behavior via brainstem premotor targets. *Nat. Neurosci.* **22**, 1477–1492 (2019).

89. Fujino, Y. *et al.* Possible functions of oxytocin/vasopressin-superfamily peptides in annelids with special reference to reproduction and osmoregulation. *J. Exp. Zool.* **284**, 401–406 (1999).

90. Bardou, I., Leprince, J., Chichery, R., Vaudry, H. & Agin, V. Vasopressin/oxytocin-related peptides influence long-term memory of a passive avoidance task in the cuttlefish, Sepia officinalis. *Neurobiol. Learn. Mem.* **93**, 240–247 (2010).

91. Van Kesteren, R. E. *et al.* Structural and functional evolution of the vasopressin/oxytocin superfamily: vasopressin-related conopressin is the only member present in Lymnaea, and is involved in the control of sexual behavior. *J. Neurosci.* **15**, 5989 LP – 5998 (1995).

92. Chen, J., Volpi, S. & Aguilera, G. Anti-apoptotic actions of vasopressin in H32 neurons involve map kinase transactivation and bad phosphorylation. *Exp. Neurol.* **211**, 529–538 (2008).

93. Schank, J. C. Early locomotor and social effects in vasopressin deficient neonatal rats. *Behav. Brain Res.* **197**, 166–177 (2009).

94. Nephew, B. C. & Bridges, R. S. Central actions of arginine vasopressin and a V1a receptor antagonist on maternal aggression, maternal behavior, and grooming in lactating rats. *Pharmacol. Biochem. Behav.* **91**, 77–83 (2008).

95. Pavan de Arruda Camargo, G. M., Saad, W. A. & de Arruda Camargo, L. A. Vasopressin and angiotensin receptors of the medial septal area in the control of mean arterial pressure induced by vasopressin. *JRAAS - J. Renin-Angiotensin-Aldosterone Syst.* **9**, 133–138 (2008).

96. Alonso, G., Gallibert, E., Lafont, C. & Guillon, G. Intrahypothalamic angiogenesis induced by osmotic stimuli correlates with local hypoxia: A potential role of confined vasoconstriction induced by dendritic secretion of vasopressin. *Endocrinology* **149**, 4279–4288 (2008).

97. Walter, R., Rudinger, J. & Schwartz, I. L. Chemistry and structure-activity relations of the antidiuretic hormones. *Am. J. Med.* **42**, 653–677 (1967).

98. Richmond, C. A. The role of arginine vasopressin in thermoregulation during fever. *The Journal of neuroscience nursing : journal of the American Association of Neuroscience Nurses* vol. 35 281–286 (2003).

99. Heinrichs, M. & Domes, G. Neuropeptides and social behaviour: effects of oxytocin and vasopressin in humans. *Progress in Brain Research* vol. 170 337–350 (2008).

100. Weingartner, H. *et al.* Effects of vasopressin on human memory functions. *Science (80-.).* **211**, 601–603 (1981).

101. Winslow, J. T., Hastings, N., Carter, C. S., Harbaugh, C. R. & Insel, T. R. A role for central vasopressin in pair bonding in monogamous prairie voles. *Nature* **365**, 545–548 (1993).

102. Goldstein, D. L. Regulation of the avian kidney by arginine vasotocin. *Gen. Comp. Endocrinol.* **147**, 78–84 (2006).

103. Kihlström, J. E. & Danninge, I. Neurohypophysial hormones and sexual behavior in males of the domestic fowl (Gallus domesticus L.) and the pigeon (Columba livia Gmel.). *Gen. Comp. Endocrinol.* **18**, 115–120 (1972).

104. Goodson, J. L. Territorial aggression and dawn song are modulated by septal vasotocin and vasoactive intestinal polypeptide in male field sparrows (Spizella pusilia). *Horm. Behav.* **34**, 67–77 (1998).

105. Baran, N. M., Peck, S. C., Kim, T. H., Goldstein, M. H. & Adkins-Regan, E. Early life manipulations of vasopressin-family peptides alter vocal learning. *Proc. R. Soc. B Biol. Sci.* **284**, 20171114 (2017).

106. Baran, N. M., Sklar, N. C. & Adkins-Regan, E. Developmental effects of vasotocin and nonapeptide receptors on early social attachment and affiliative behavior in the zebra finch. *Horm. Behav.* **78**, 20–31 (2016).

107. Butler, D. G. & Snitman, F. S. Renal responses to mesotocin in Western painted turtles compared with the antidiuretic response to arginine vasotocin. *Gen. Comp. Endocrinol.* **144**, 101–109 (2005).

108. Hattori, T. & Wilczynski, W. Comparison of arginine vasotocin immunoreactivity differences in dominant and subordinate green anole lizards. *Physiol. Behav.* **96**, 104–107 (2009).

109. Figler, R. A., MacKenzie, D. S., Owens, D. W., Licht, P. & Amoss, M. S. Increased levels of arginine vasotocin and neurophysin during nesting in sea turtles. *Gen. Comp. Endocrinol.* **73**, 223–232 (1989).

110. Mahmoud, I. Y., Cyrus, R. V., McAsey, M. E., Cady, C. & Woller, M. J. The role of arginine vasotocin and prostaglandin F 2α on oviposition and luteolysis in the common snapping turtle Chelydra serpentina. *Gen. Comp. Endocrinol.* **69**, 56–64 (1988).

111. Guillette, L. J. Stimulation of parturition in a viviparous lizard (Sceloporus jarrovi) by arginine vasotocin. *Gen. Comp. Endocrinol.* **38**, 457–460 (1979).

112. Boyd, S. K. Arginine vasotocin facilitation of advertisement calling and call phonotaxis in bullfrogs. *Horm. Behav.* **28**, 232–240 (1994).

113. Moore, F. L., Wood, R. E. & Boyd, S. K. Sex steroids and vasotocin interact in a female amphibian (Taricha granulosa) to elicit female-like egg-laying behavior or male-like courtship. *Horm. Behav.* **26**, 156–166 (1992).

114. Moore, F. L. & Miller, L. J. Arginine vasotocin induces sexual behavior of newts by acting on cells in the brain. *Peptides* **4**, 97–102 (1983).

115. Thompson, R. R. & Moore, F. L. Vasotocin stimulates appetitive responses to the visual and pheromonal stimuli used by male roughskin newts during courtship. *Horm. Behav.* **38**, 75–85 (2000).

116. Salek, S. J., Sullivan, C. V. & Godwin, J. Arginine vasotocin effects on courtship behavior in male white perch (Morone americana). *Behav. Brain Res.* **133**, 177–183 (2002).

117. Semsar, K., Kandel, F. L. M. & Godwin, J. Manipulations of the AVT system shift social status and related courtship and aggressive behavior in the bluehead wrasse. *Horm. Behav.* **40**, 21–31 (2001).

118. Braida, D. *et al.* Neurohypophyseal hormones manipulation modulate social and anxiety-related behavior in zebrafish. *Psychopharmacology (Berl).* **220**, 319–330 (2012).

119. Hiraoka, S., Ando, H., Ban, M., Ueda, H. & Urano, A. Changes in expression of neurohypophysial hormone genes during spawning migration in chum salmon, Oncorhynchus keta. *J. Mol. Endocrinol.* **18**, 49–55 (1997).

120. Gilchriest, B. J., Tipping, D. R., Levy, A. & Baker, B. I. Diurnal changes in the expression of genes encoding for arginine vasotocin and pituitary pro-opiomelanocortin in the rainbow trout (Oncorhynchus mykiss): Correlation with changes in plasma hormones. *J. Neuroendocrinol.* **10**, 937–943 (1998).

121. Le Mevel, J. C., Pamantung, T. F., Mabin, D. & Vaudry, H. Effects of central and peripheral administration of arginine vasotocin and related neuropeptides on blood

pressure and heart rate in the conscious trout. *Brain Res.* **610**, 82–9 (1993).

122. Henderson, I. W. & Wales, N. A. M. Renal diuresis and antidiuresis after injections of arginine vasotocin in the freshwater eel (Anguilla anguilla L.). *J. Endocrinol.* **61**, 487–500 (1974).

123. Mayasich, S. A. & Clarke, B. L. *Characterization of the vasotocin neuropeptide hormone receptor system in the sea lamprey (Petromyzon marinus) IN PARTIAL FULFILLMENT OF THE REQUIREMENTS FOR THE DEGREE OF DOCTOR OF PHILOSOPHY.* (2015).

124. Bentley, P. J. & Folley, B. K. The effects of hormones on the carbohydrate metabolism of the lamprey (Lampetra Fluviatilis). *J. Endocrinol.* **31**, 127–137 (1965).

125. Proux, J. P. *et al.* Identification of an arginine vasopressin-like diuretic hormone from Locusta migratoria. *Biochem. Biophys. Res. Commun.* **149**, 180–186 (1987).

126. Aikins, M. J. *et al.* Vasopressin-like peptide and its receptor function in an indirect diuretic signaling pathway in the red flour beetle. Tribolium project View project Mechanisms of K+ transport across basolateral membranes of principal cells in Malpighian tubules of the yellow fever mosquito, Aedes aegypti View project Vasopressin-like peptide and its receptor function in an indirect diuretic signaling pathway in the red flour beetle. (2018) doi:10.1016/j.ibmb.2008.04.006.
