# Supplementary material for: Universal nomenclature for oxytocin–vasotocin ligand and receptor families
Source: Nature. 2021 Apr 28;592(7856):747–55. doi: 10.1038/s41586-020-03040-7 (PMC8081664; doi:10.1038/s41586-020-03040-7)
Supplement: Supplementary file 2 — Reporting Summary [file 41586_2020_3040_MOESM2_ESM.pdf]

nature research

Corresponding author(s): Jarvis, ED.; Theofanopoulou C.

Last updated by author(s): 2020/2/11

Reporting Summary

Nature Research wishes to improve the reproducibility of the work that we publish. This form provides structure for consistency and transparency in reporting. For further information on Nature Research policies, see Authors & Referees and the Editorial Policy Checklist .

Statistics

For all statistical analyses, confirm that the following items are present in the figure legend, table legend, main text, or Methods section.

n/a	Confirmed	
☐	☒	The exact sample size (*n*) for each experimental group/condition, given as a discrete number and unit of measurement
☐	☒	A statement on whether measurements were taken from distinct samples or whether the same sample was measured repeatedly
☐	☒	The statistical test(s) used AND whether they are one- or two-sided<br>*Only common tests should be described solely by name; describe more complex techniques in the Methods section.*
☐	☒	A description of all covariates tested
☐	☒	A description of any assumptions or corrections, such as tests of normality and adjustment for multiple comparisons
☐	☒	A full description of the statistical parameters including central tendency (e.g. means) or other basic estimates (e.g. regression coefficient) AND variation (e.g. standard deviation) or associated estimates of uncertainty (e.g. confidence intervals)
☐	☒	For null hypothesis testing, the test statistic (e.g. *F*, *t*, *r*) with confidence intervals, effect sizes, degrees of freedom and *P* value noted<br>*Give P values as exact values whenever suitable.*
☒	☐	For Bayesian analysis, information on the choice of priors and Markov chain Monte Carlo settings
☒	☐	For hierarchical and complex designs, identification of the appropriate level for tests and full reporting of outcomes
☒	☐	Estimates of effect sizes (e.g. Cohen's *d*, Pearson's *r*), indicating how they were calculated

Our web collection on statistics for biologists contains articles on many of the points above.

Software and code

Policy information about availability of computer code

Data collection

We used 35 vertebrate and 4 invertebrate species' genomes, whose IDs and GenBank assembly accession numbers can be found in Supplementary Table 1. All the NCBI/Ensembl/Gene IDs of the genes we studied can be found in Supplementary Tables 3 (S3a-S3e) and 4. All the gene sequences used for the phylogenetic trees can be found here: https://github.com/constantinatheo/otvt.

Data analysis

We used the BLAT(available in the UCSC genome browser and alignment; last update: Nov 9, 2018) /BLAST(v2.9.0+), SynMap2, GeVo (no version available; last update: Sept 16, 2019) and SynFind (no version available; last update Sept 16, 2019) tools for the synteny analyses. For the sea lamprey (gPmar1.0.9), we used the BLAST, Genome Browser and Gene Search tools available in https://genomes.stowers.org/organism/Petromyzon/marinus. For the amphioxus (B. floridae v2.0), we used the BLAST and Gene Browser tools available in https://genome.jgi.doe.gov/Brafl1/Brafl1.home.html. SynFind results were further parsed using biomaRt (v3.10) and visualized using a custom an R script (v3.6.1) (https://github.com/ggedman/OT_VT_synteny).

We quantitatively searched for DNA transposable elements (TEs) around the OT and VT region in the human and chimpanzee genomes using the RepeatMasker tool (last update: March 20, 2015) in the UCSC Genome Browser (http://genome.ucsc.edu/) and we obtained information for each specific TE via Dfam 2.0. We calculated the GC content using ENDMEMO (no version or last update date available) (http://www.endmemo.com/bio/gc.php/). We aligned the introns of human OT and VT in all possible combinations using DIALIGN (v2.2.1) and compared the length of the introns with the higher identity (first intron of OT vs. first intron of VT) using the Serial Cloner v.2.6 (http://serialbasics.free.fr/Serial_Cloner.html).

To analyze conserved non-coding RNA synteny around the OTR-VTRs, we looked for them in alignments in all the species studied in Ensembl (v95), in the miRbase (http://www.mirbase.org/; miRbase 22 release), and the miRviewer database (last update: Feb 28, 2012).

The exonic gene sequences were aligned with MAFFT (v7) under the E-INS-i parameter. From this alignment, a Phylogenetic Maximum Likelihood tree was generated using RAXML's (v8.2.10) GTRGAMMA model with 1000 replicates.

The protein-coding Maximum Likelihood phylogenetic tree was constructed with the 'Gene tree' tool in Ensembl (v95) (Gene Tree ID: ENSGT00760000119156): gene trees were constructed using one representative amino acid sequence for every gene in every species using TreeFAM (v9) and TreeBeST (v1.9.2) pipeline in an Ensembl (v95) package.

All relevant references and links are available in the 'Methods' section.

For manuscripts utilizing custom algorithms or software that are central to the research but not yet described in published literature, software must be made available to editors/reviewers. We strongly encourage code deposition in a community repository (e.g. GitHub). See the Nature Research guidelines for submitting code & software for further information.

Data

Policy information about availability of data

All manuscripts must include a data availability statement. This statement should provide the following information, where applicable:

- Accession codes, unique identifiers, or web links for publicly available datasets
- A list of figures that have associated raw data
- A description of any restrictions on data availability

All the data and codes used in this study can be found in the Suppl.Tables_Theofanopoulou excel document and in the following depositories: https://github.com/constantinatheo/otvt; https://github.com/ggedman/OT_VT_synteny.

Field-specific reporting

Please select the one below that is the best fit for your research. If you are not sure, read the appropriate sections before making your selection.

☒ Life sciences ☐ Behavioural & social sciences ☐ Ecological, evolutionary & environmental sciences

For a reference copy of the document with all sections, see nature.com/documents/nr-reporting-summary-flat.pdf

Life sciences study design

All studies must disclose on these points even when the disclosure is negative.

Sample size

For our microsynteny analyses we used 35 vertebrate and 4 invertebrate species' genomes. These included newly re-sequenced species (pale spear-nosed bat, platypus, Anna's hummingbird, zebra finch, blunt-snouted clingfish) with long-read (Pacbio) and long-range scaffolding (Bionano optical maps, Hi-C and 10X link reads) technologies generated by the Vertebrate Genomes Project (VGP; https://vertebrategenomesproject.org). We selected the species we used in order to represent all major vertebrate lineages. There was not a sample-size calculation that led to the decision of the sample size. Sample sizes do not apply in these cases in the way they apply to other experiments, where, for example, many individuals are tested in the same set up to replicate the result. In the case of a genomics' study like ours, this would be possible only if there were different high-level assemblies of the same species, but still it would be very rare (extremely costly) to reach a statistically sufficient amount of individuals.

For our macrosynteny chromosomal-scale analyses we used genomes that have been sequenced at a chromosome-level (japanese medaka, zebrafish, chicken, frog, human) to compare with the superscaffold-level assembly of sea lamprey and the scaffold-level assembly of inshore hagfish. We chose these species' genomes in order to represent chromosome-level assemblies from as many vertebrate lineages as possible (we represented teleost fish, birds, amphibians and mammals) when compared to the sea lamprey (lineage: lampreys) and the inshore hagfish (lineage: hagfishes). In the rest of the lineages that were not represented (holostean fish, sharks, coelacanths, reptiles) there are not any chromosome-level assemblies available yet. For the purpose of the study it was critical to include chromosome-level assemblies (quality), instead of as many assemblies as possible (quantity), that would not serve to resolve the evolutionary question.

For the protein-coding phylogeny we used all the species' genomes included in the Ensembl (v95) database.
For the exonic phylogeny we used the longest read-sequences available from species representing all major vertebrate lineages (human and mouse for mammals, chicken for birds, turtle and lizard for reptiles, frog for amphibians, coelacanth for coelacanths, zebrafish for teleost fish, spotted gar for holostean fish, elephant shark for sharks, sea lamprey for lampreys, inshore hagfish for hagfishes). The sample size is sufficient in terms of quantity (since all major vertebrate lineages are represented) and quality (we used only long-read sequences).

Data exclusions

We did not exclude any genomes of species that would have contributed further to the understanding of the evolution of the OT-VT ligands and the OTR-VTR receptors.
In our exonic phylogenetic tree, any non-lamprey OTR-VTR sequences less than 1000 bp (i.e. incomplete) were excluded, as alignments on short sequences may lack power to resolve species' relationships, resulting in weakly supported gene trees. Because of the lamprey's basal phylogenetic position, all lamprey OTR-VTRs (754 bp and longer) were included.

Replication

We replicated our microsynteny findings on the distribution of the OTR-VTR receptors in vertebrates, using macrosynteny (up to 100-gene window, chromosome-scale), phylogeny (exonic and protein coding trees) and ancestral (mapping our regions of interest back to putative ancestral vertebrate or chordate chromosomes) analyses.

Randomization

Randomization was not relevant in this study. We used in all the analyses the genomes with the highest-quality assemblies.

Blinding	Our tests were blind in that we had not assigned specific names to the genes before our synteny analyses showed clearly which gene is orthologous to which.

Reporting for specific materials, systems and methods

We require information from authors about some types of materials, experimental systems and methods used in many studies. Here, indicate whether each material, system or method listed is relevant to your study. If you are not sure if a list item applies to your research, read the appropriate section before selecting a response.

Materials & experimental systems

n/a	Involved in the study	
☒	☐	Antibodies
☒	☐	Eukaryotic cell lines
☒	☐	Palaeontology
☒	☐	Animals and other organisms
☒	☐	Human research participants
☒	☐	Clinical data

Methods

n/a	Involved in the study	
☒	☐	ChIP-seq
☒	☐	Flow cytometry
☒	☐	MRI-based neuroimaging
